# Towards improved butanol production through targeted genetic modification of *Clostridium pasteurianum*

**DOI:** 10.1016/j.ymben.2017.01.009

**Published:** 2017-03

**Authors:** Katrin M. Schwarz, Alexander Grosse-Honebrink, Kamila Derecka, Carlo Rotta, Ying Zhang, Nigel P. Minton

**Affiliations:** Clostridia Research Group, BBSRC/EPSRC Synthetic Biology Research Centre (SBRC), University of Nottingham, University Park, Nottingham NG7 2RD, UK

**Keywords:** *Clostridium pasteurianum*, Rex, HydA, DhaBCE, Butanol, 1,3-propanediol

## Abstract

Declining fossil fuel reserves, coupled with environmental concerns over their continued extraction and exploitation have led to strenuous efforts to identify renewable routes to energy and fuels. One attractive option is to convert glycerol, a by-product of the biodiesel industry, into *n-*butanol, an industrially important chemical and potential liquid transportation fuel, using *Clostridium pasteurianum.* Under certain growth conditions this *Clostridium* species has been shown to predominantly produce *n*-butanol, together with ethanol and 1,3-propanediol, when grown on glycerol. Further increases in the yields of *n*-butanol produced by *C. pasteurianum* could be accomplished through rational metabolic engineering of the strain. Accordingly, in the current report we have developed and exemplified a robust tool kit for the metabolic engineering of *C. pasteurianum* and used the system to make the first reported in-frame deletion mutants of pivotal genes involved in solvent production, namely *hydA* (hydrogenase), *rex* (Redox response regulator) and *dhaBCE* (glycerol dehydratase). We were, for the first time in *C. pasteurianum*, able to eliminate 1,3-propanediol synthesis and demonstrate its production was essential for growth on glycerol as a carbon source. Inactivation of both *rex* and *hydA* resulted in increased *n-*butanol titres, representing the first steps towards improving the utilisation of *C. pasteurianum* as a chassis for the industrial production of this important chemical.

## Highlights

• A comprehensive molecular tool set for *Clostridium pasteurianum*.
• Abolishment of by-product (1,3-propanediol) formation by *dhaBCE* KO.
• First reported deletion of main hydrogenase HydA in *Clostridium* spp.
• Inactivation of *rex* and *hydA* leads to increased *n*-butanol titres.

## 1 Introduction

Declining fossil fuel reserves, coupled with environmental concerns over their continued extraction and exploitation have led to strenuous efforts to identify renewable routes to energy and fuels. The two most commonly used biofuels are bioethanol and biodiesel (REN21, 2015). Between 2004 and 2014 biodiesel production has increased more than twelvefold, from 2.4 billion litres/year to 29.7 billion litres/year (REN21, 2015). For every ton of biodiesel produced through the transesterification of vegetable oils and animals fats with short-chain alcohols (most commonly methanol) 100 kg of crude glycerol (10% [w/w]), or glycerine, are formed as a by-product. As a consequence, glycerol availability has increased significantly (Johnson and Taconi, 2007, Yang et al., 2012). The glycerol formed is almost always used in its refined or purified form to make a multitude of products, including cosmetics, pharmaceuticals and food and beverages (Quispe et al., 2013). The glut of glycerine that has resulted from the biodiesel industry has impacted crude and refined glycerol prices. Economically, crude glycerol has shifted from by-product to waste-product (Kerr et al., 2011, Quispe et al., 2013). With the associated disposal costs, ways to convert crude glycerol into valuable products are becoming increasingly important (Yazdani and Gonzalez, 2007). Currently crude glycerol is utilised as a supplement for animal feeds, reacting agent in chemical catalytic procedures and as a feedstock for the biological production of chemicals and fuels (Yang et al., 2012). The high degree of reduction of its carbon atoms (Clomburg and Gonzalez, 2013), its wide availability and low cost have made glycerol an attractive feedstock for biorefinery (Werpy et al., 2004; Cherubini, 2009; Dobson et al., 2012), especially for anaerobic fermentation processes (Yazdani and Gonzalez, 2007).

*Clostridium pasteurianum* is, unlike other well-studied clostridia, capable of converting glycerol directly into the value-added chemicals *n*-butanol and 1,3-propanediol (PDO) (Biebl, 2001, Jensen et al., 2012, Malaviya et al., 2012). Butanol is an attractive biofuel as it offers a high energy density, low water solubility, low vapour pressure and good blending abilities and can be used in regular combustion engines without the need of modification (Dürre, 2007). The organic compound PDO is used as a precursor for the production of useful polymers such as polyesters, polyurethans and polyethers. The polyester polytrimethylene terephthalate (PTT) accounts for 90.0% of the total PDO market which is expected to be worth $621.2 million by 2021 (Market and Market, 2015). It shares many of the features of its polyester counterpart polybutylene terephthalate (PBT) and polyethylene terephthalate (PET) but offers higher tensile and flexural strengths and stiffness (Zhang, 2004). In addition, PDO is used in composites, adhesives, laminates, coatings, moldings, aliphatic polyesters and antifreeze (Liu et al., 2010).

**Fig. 1:**
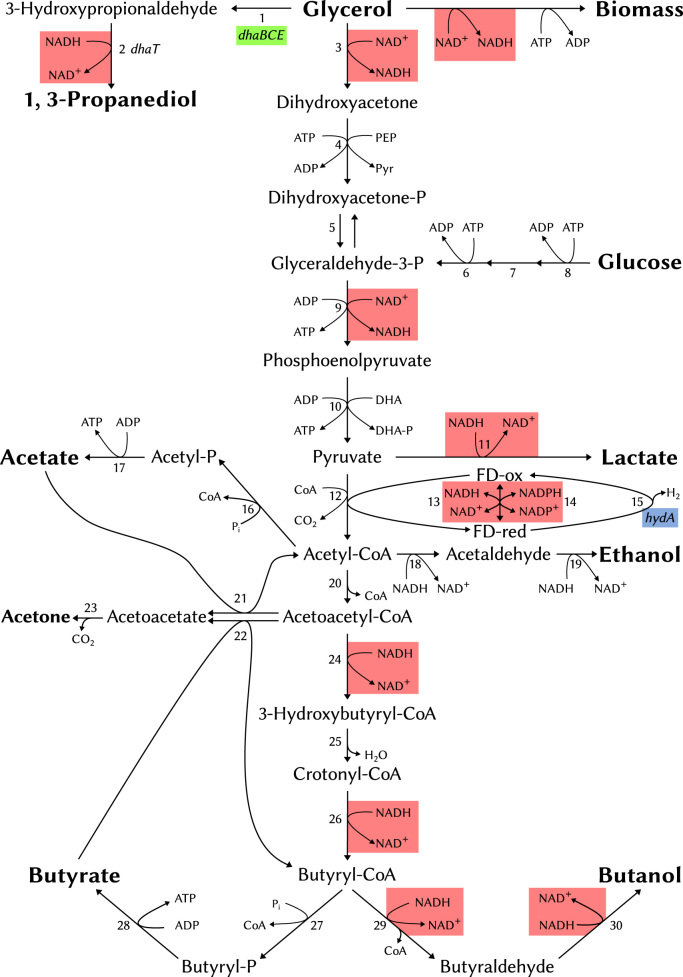
Central metabolic energy pathway in *C. pasteurianum* from glucose and glycerol. Figure based on Malaviya et al. (2012) and Biebl (2001). The knocked out genes (*hydA, dhaBCE*) and the NADH/NAD^+^ utilising pathways putatively influenced by Rex are highlighted. Other enzymes involved in the central energy pathway are numbered as follows: 1, glycerol dehydratase; 2, 1,3-propanediol oxydoreductase. 3, glycerol-3-phosphate dehydrogenase; 4, dihydroxyacetone kinase; 5, triose-phosphate isomerase; 6, phosphofructokinase; 7, phosphoglucose isomerase; 8, hexokinase; 9, glyceraldehyde-3-phosphate dehydrogenase; 10, pyruvate kinase; 11, lactate dehydrogenase; 12, pyruvate-ferredoxin oxidoreductase; 13, ferredoxin-NADP reductase; 14, NADPH-ferredoxin oxidoreductase; 15, ferredoxin hydrogenase; 16, phosphate acetyltransferase; 17, acetate kinase; 18, acetaldehyde dehydrogenase; 19, ethanol dehydrogenase; 20, thiolase; 21, acetoacetyl-CoA: acetate:CoA transferase; 22, acetoacetyl-CoA: butyrate:CoA transferase; 23, acetoacetate decarboxylase; 24, β -hydroxybutyryl-CoA dehydrogenase; 25, crotonase; 26, butyryl-CoA dehydrogenase; 27, phosphotransbutyrylase; 28, butyrate kinase; 29, butaraldehyde dehydrogenase; 30, butanol dehydrogenase.

Under certain growth conditions *C. pasteurianum* has been shown (Dabrock et al., 1992, Jensen et al., 2012, Moon et al., 2011; Taconi et al., 2009) to predominantly produce *n*-butanol, ethanol and PDO with trace amounts of organic acids (see Fig. 1). In optimized batch cultures up to 17 g/l *n*-butanol have been produced from glycerol, in pH auxostat cultures around 7 g/l PDO (Biebl, 2001). Recently, a *C. pasteurianum* mutant strain was generated producing 17.8 g/l *n*-butanol and 8.7 g/l PDO from refined glycerol (Malaviya et al., 2012). In comparison, engineered *C. acetobutylicum* and *C. beijerinckii* mutant strains have been described to produce 18.9 g/l and 20.9 g/l of butanol (Chen and Blaschek, 1999; Jang et al., 2012). Further increases in yields of *n*-butanol produced by *C. pasteurianum* could be accomplished through rational metabolic engineering of the strain. Accordingly, in the present study we have developed the requisite gene modification systems through the implementation of our previously described roadmap for gene tool development (Minton et al., 2016), and then used the developed allelic exchange vectors to make in-frame deletions of *spo0A* (for exemplification purposes) and a selection of mutants likely to affect solvent yields, namely, *dhaBCE* (encoding glycerol dehydratase), *rex* (coding for the redox responsive repressor Rex) and *hydA* (encoding an iron-coupled hydrogenase).

## 2 Methods

### 2.1 Bacterial strains, growth and maintenance conditions

Bacterial strains used are listed in Table 1. *E. coli* was grown aerobically at 30 °C or 37 °C in Luria-Bertani (LB) broth or agar supplemented with 25 µg/ml chloramphenicol (LB_Cm25_) or 100 µg/ml kanamycin (LB_Km100_), respectively. *C. pasteurianum* was routinely grown at 37 °C in an anaerobic workstation (Don Whitley, Yorkshire, UK) in 2x YTG broth (16 g/l tryptone, 10 g/l yeast extract, 5 g/l NaCl, 5 g/l glucose, pH 6.2) or on RCM agar (Oxoid Ltd) supplemented with 15 µg/ml thiamphenicol (2x YTG_Tm15_, RCM_Tm15_), 20 µg/ml erythromycin (2x YTG_Em20_, RCM_Em20_) or 40 µg/ml uracil (2x YTG_Ura40_, RCM_Ura40_), if required. Selections using 5-fluoroorotic acid (FOA, 600 µg/ml; Sigma-Aldrich, Dorset, UK) and uracil (5 µg/ml) were carried out in modified clostridial basal medium containing 0.5% (w/v) CaCO_3_ and 5% (w/v) glucose (CBM-S) (Steiner et al., 2012) or on standard clostridial basal medium (CBM) agar (O'Brien and Morris, 1971). All solidified media contained 1.5% [w/v] agar. Solvent/ acid profiling was undertaken in CGM (Sandoval et al., 2015), Biebl medium (Biebl, 2001) or 2x YT media supplemented with 60 g/l glycerol or glucose. Biebl medium was additionally supplemented with 1 g/l yeast extract for glycerol fermentations Table 2.

### 2.2 Standard molecular biology techniques

Standard plasmids used in this study are detailed in Table 1. Electroporation of *E. coli* was carried out using a Gene-Pulsar (Bio-Rad, Hemel Hempstead, UK) in agreement with the manufacturer's instructions. Plasmid DNA and genomic DNA was isolated using a QIAprep spin miniprep or a DNeasy blood and tissue kit (Qiagen, Manchester, UK), respectively. Restriction digest by endonucleases, ligation reactions and agarose gel electrophoresis were performed according to the manufacturer's instructions. Restriction endonucleases were purchased from ThermoFisher Scientific (Loughborough, UK) and a LigaFast™ Rapid DNA Ligation kit from Promega (Southampton, UK). PCR was carried out using the BIO-X-ACT™ Short Mix (Bioline Reagents, London, UK) in accordance with the manufacturer's instructions. Site-directed mutagenesis (SDM) was carried out using the Quik Change II Site-Directed Mutagenesis Kit (Agilent Technologies, Stockport, UK). Oligonucleotides were ordered from Eurofins Genomics (Ebersberg, Germany) and are given in Supplementary Table S1. Sanger sequencing was carried out by Source BioSciences (Nottingham, UK). Synthetic DNA fragments were ordered either from Biomatik (Cambridge, Ontario/Canada) or GeneArt® Gene Synthesis (Life Technologies, Paisley, UK).

### 2.3 Plasmid methylation and plasmid transfer into *C. pasteurianum*

Plasmid DNA for the transformation into *C. pasteurianum* strain was *in vivo* methylated by propagation in the *dam*^+^, *dcm*^+^ *E. coli* host CR1, an *E. coli* Top10 strain harbouring the plasmid pCR1, comprising a RSF1030-derived RSF origin of replication (Som and Tomizawa, 1982, Novagen, Merck KGaA, Darmstadt, Germany), a kanamycin resistance marker and a gene encoding the M.BepI methyltransferase of *Brevibacterium epidermidis* under the transcriptional control of the *C. sporogenes fdx* promoter (P_*Csp fdx*_). Alternatively, *in vitro* methylation was performed by incubation of plasmid DNA with CpG methyltransferase (M.SssI; NEB, Hitchin, UK), according to the manufacturer's instructions. Methylated plasmids 0.5–5 μg DNA) were electroporated into *C. pasteurianum* as detailed in Supplementary Information.

### 2.4 Construction of Allele-Coupled Exchange vectors

**Fig. 2:**
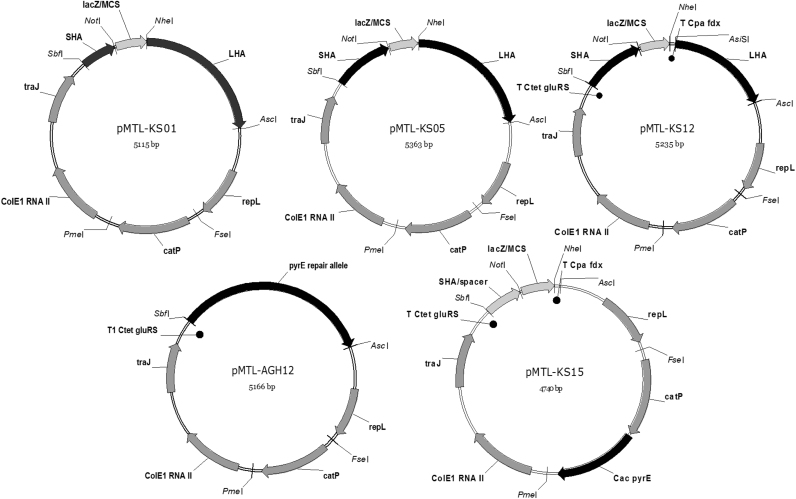
Plasmid maps of major plasmids used in this study.

All Allele-Coupled Exchange (ACE) vectors generated in this study were based on the modular plasmid pMTL85151 (Heap et al., 2009) and conform to previous design principles (Heap et al., 2012). The *pyrE* ACE KO vector (pMTL-KS01) used to generate a ∆*pyrE* mutant (strain CRG4273) of *C. pasteurianum* DSM 525 was based on pMTL-JH12 (Heap et al., 2012). The requisite *pyrE* KO cassette comprised a long (1200 bp) right homology arm (RHA) homologous to a region downstream of *pyrE* and a short (300 bp) left homology arm (LHA) composed of an internal portion (nt 35–334) of the *pyrE* gene (full details in Supplementary Information). The *pyrE* ACE correction (pMTL-AGH12, Fig. 2) and complementation (pMTL-KS12, Fig. 2) vectors were constructed equivalent to pMTL-YN1/YN2 and pMTL-YN1C/YN2C (Ng et al., 2013). Plasmid pMTL-AGH12 was generated through the PCR amplification of a 1748-bp fragment comprising the *pyrE* nucleotides 35–582 (RHA) and a contiguous region 1200-bp downstream of its stop codon (LHA) using primers AGH0001_pyrE_Fw and AGH0001_pyrE_Rev (Supplementary Table S1) and CRG4111 genomic DNA. Following amplification, the DNA product generated was cleaved with *Sbf*I and *Asc*I, and inserted between the *Sbf*I and *Asc*I sites of pMTL-KS10 (More information in Supplementary Information). Plasmid pMTL-KS12 (Fig. 2) was essentially the same as pMTL-AGH12, except in this case the RHA and LHA were separated by a segment of DNA encompassing a *lacZ’*-encoding region containing a MCS region. In contrast to pMTL-YN1C/YN2C, however, the RHA was followed by a 77-bp fragment encoding the *C. tetani* E88 glutamyl-tRNA synthetase terminator (T_*Ctet gluRS*_), while the LHA was preceded by a 42-bp DNA region encompassing the *C. pasteurianum* transcriptional terminator of the ferredoxin gene (T_*Cpa fdx*_). Full details of the construction of pMTL-KS12 is given in Supplementary Information.

For the generation of the complementation vectors target genes including their native promoter were PCR amplified using CRG4111 genomic DNA as template and a gene specific oligonucleotide pair (Fw and Rev) encompassing 5’-*Not*I and a 3’-*Nhe*I recognition sites (see Supplementary Table S1). Following amplification, the DNA fragment was digested with *Not*I and *Nhe*I, purified and ligated into appropriately cleaved pMTL-KS12. Full details of the construction of the complementation vectors is given in Supplementary Information.

### 2.5 Construction of allelic exchange KO vectors

All generated allelic exchange vectors are based on the modular plasmid pMTL85151 (Heap et al., 2009) designed in accordance with Ng et al. (2013). For the marker-less, in-frame deletion of genes in strain CRG4273 (Table 1), the *pyrE* gene of *C. acetobutylicum* was employed as a heterologous counter (negative) selection marker. Therefore, the *catP*-*pyrE* module of the plasmid pMTL-AMH101 (Liew et al., 2017) was cloned as a *Fse*I-*Pme*I-fragment into the equivalent sites of pMTL-KS08. The resulting plasmid, pMTL-KS15 (Table 1, Fig. 2), carried strong transcriptional terminators (5’ T_*Ctet gluRS*_, 3’ T_*Cpa fdx*_) that flanked the site of insertion (between *Sbf*I and *Nhe*I) of the KO cassette. Cassettes were generated by SOE (splicing by overlap extension) PCR as described previously (Ng et al., 2013) and cloned into pMTL-KS15 *via Sbf*I/*Nhe*I sites. Full details of the construction of AE KO plasmids can be found in Supplementary Information.

### 2.6 Allele‐Coupled Exchange procedure

Allele-Coupled Exchange (ACE) vectors were transformed into *C. pasteurianum* DSM 525-H1Δ*pyrE* (CRG4273) and plated onto RCM_Tm15_ agar. Putative single crossover integrants were identified as faster growing, larger colonies (Cartman et al., 2012). These were restreaked onto RCM_Tm15_ agar and PCR screened for the presence of single crossovers, using primers KS004_Cpa_pyrE_gen_Fw and KS007_ 85151_LHA_Rev2 (Supplementary Table S1). In the case of *pyrE* KO using pMTL-KS01, clones confirmed as single crossovers were re-streaked onto CBM_FOA600, Ura5_ agar plates and incubated for 24- 28 h. Colonies that developed represented putative double crossover mutants. These were patch plated onto CBM, CBM_Tm15U5_ and CBM_U5_ agar plates to identify double crossover; uracil auxotrophs that had lost the excised plasmid grow only on the latter media. Deletion mutants were verified by Sanger sequencing of the PCR amplified DNA fragment generated using the above primer pair.

The procedure was the same for restoration of the *pyrE* locus to wild type using the *pyrE* ACE correction vector (pMTL-AGH12) and the *pyrE* ACE complementation vectors (pMTL-KS12) carrying functional copies of the gene to be complemented, but in this case the faster growing, single crossover integrants were re-streaked twice onto RCM_Tm15_ agar plates to purify and incubated for 16- 24 h. Single colonies were then restreaked onto CBM agar plates, and replica plated onto RCM_Tm15_ agar plates to check for plasmid loss. Following, successive re-streaking (3- 5 times) onto fresh CBM and RCM_Tm15_, clones were identified that exhibited strong growth on CBM but not on RCM_Tm15_ agar plates. Restoration of the *pyrE* locus to WT was confirmed by Sanger sequencing of the DNA fragment PCR amplified using primers KS004_Cpa_pyrE_gen_Fw and KS004_Cpa_pyrE_gen_Rev (Supplementary Table S1).

### 2.7 Allelic exchange KO procedure

For the construction of specific gene deletions (*spo0A, dhaBCE, hydA* and *rex*) by allelic exchange, appropriate KO vectors based on pMTL-KS15 were transformed into the *pyrE* deletion strain CRG4273 (Table 1) and plated on RCM_Tm15_ agar. Following 48 h incubation, faster growing colonies were re-streaked twice onto RCM_Tm15_ agar plates and their identity as single crossover integrants confirmed by Sanger sequencing of the PCR amplified DNA fragment using appropriate primer pairs. Confirmed single crossover mutants were grown overnight in 5 ml CBMS_FOA600, Ura5_ broth to allow the double crossover to occur, centrifuged (10 min, 8500*g*, RT) and re-suspended in 250 µl PBS. A 100 µl aliquot of the suspended cells was serially diluted (up to 10^−7^) in PBS and 100 µl of each dilution plated onto RCM agar plates. After a 24 h incubation, 50 single, faster growing colonies were selected and re-streaked in the indicated order onto RCM_Tm15_, CBM and RCM agar plates. Colonies, which lost the plasmid and either reverted back to the WT or carried the desired deletion exhibited no growth on RCM_Tm15_ and CBM agar plates but grew on RCM agar plates. These re-streaks were subjected to colony PCR, using gene specific primers that flanked the intended deletion, and the amplified DNA fragment subjected to Sanger sequencing to confirm the expected genotype. The *pyrE* deletion of verified mutants was restored to WT using the ACE plasmid pMTL-AGH12 (Fig. 2).

### 2.8 Next-generation sequencing and resequencing analysis

Illumina sequencing of genomic DNA was done by the Deep Seq: Next Generation Sequencing Facility (University of Nottingham, UK) using a MiSeq Illumina system (Illumina, USA). Paired-end reads were mapped against the published *C. pasteurianum* DSM 525 (strain CRG4091) genome (Poehlein et al., 2015) CLC Genomics Workbench 8.0.2 (Qiagen, DK). Single nucleotide polymorphisms (SNPs) were analysed using the basic variant caller in CLC Genomics Workbench 8.0.2 (Qiagen, DK).

### 2.9 Fermentation in serum flasks

To analyse butanol production, strains were grown either in 50 ml CGM (Sandoval et al., 2015), Biebl medium (Biebl, 2001) or 2x YTG broth in serum bottles at 37 °C with a starting pH of 6.2. Media was supplemented with 60 g/l glucose or glycerol as carbon source. Biebl medium was additionally supplemented with 1 g/l yeast extract for glycerol fermentations. Cultures were initiated in an anaerobic workstation (Don Whitley, Yorkshire, UK) by inoculating and re-suspending several colonies from CBM agar plates into 10 ml CGM, Biebl or 2x YTG broth containing 60 g/l glucose. After overnight incubation, 1 ml of culture was used to inoculate 20 ml CGM or 2x YTG or 35 ml Biebl broth supplemented with 60 g/l glucose or glycerol and incubated for 6 h prior to diluting up to 10^−3^ and incubating in fresh media and growing overnight. Those cultures that were in mid-exponential phase were used to inoculate 50 ml CGM, Biebl or 2x YTG broth supplemented with 60 g/l glucose or glycerol to a starting OD_600_ of 0.05 (CGM, 2x YTG, Biebl_glucose_) or 0.075 (Biebl_glycerol_). At this point, serum bottles were removed from the anaerobic workstation and incubated 37 °C for up to 48 h. The OD_600_ and pH were monitored and samples for product analysis taken every 3 h initially then at 24 h and close to the end of fermentation. All culturing was carried out in triplicate.

### 2.10 pH-controlled batch fermentation

To enable optimum butanol production, pH-controlled batch fermentations were carried out in a Multifors 2 parallel reactor system (Infors UK, Reigate, UK) containing 350 ml Biebl broth supplemented with 60*g*/l glucose or glycerol and 1*g*/l yeast extract were incubated at 37 °C, 250 rpm and continuous sparging with sterile N_2_ (1 l/min). The initial pH of the medium was 6.0. Following inoculation the pH was held above 6.0 by the automatic addition of 4 M KOH. Pre-cultures were grown as described above for the serum flask fermentation, with the exception, that the volume of the final 16 h dilution series was 35 ml. The main fermentation broth was inoculated to a starting OD_600_ of 0.75. Batch fermentations were run for 48 h. Growth was monitored through online optical density (OD) as well as through the external measurement of triplicate samples employing a BioMate 3 spectrophotometer (Thermo Fisher Scientific, Loughborough, UK). Samples for solvent analysis were taken at the same time. Additionally the pH, redox potential and temperature of the culture broth were monitored online.

To enable optimum sporulation, pH-controlled batch fermentations were carried out at 37 °C, 200 rpm, with continuous sparging of sterile N_2_ (0.02 ml/ml) in the same Multifors 2 parallel reactor system (Infors UK, Reigate, UK) containing 400 ml of CBM broth comprising no CaCO_3_ but 6% (w/v) glucose. To set up the pH-controlled batch fermentation, the strain of interest was streaked from -80 °C stocks onto RCM agar plates, incubated overnight at 37 °C and subsequently used to inoculate 10 ml CBM-S broth containing 6% (w/v) glucose. The 10 ml culture was grown overnight and used to inoculate 50 ml CBM-S broth at a 2% (v/v) inoculum. A 50 ml aliquot of this pre-culture was used to start the pH-controlled batch culture at an inoculum of 6.5% (v/v). The pH was allowed to drop from 6.5 to 5.5, before being maintained at 5.5 by the addition of 4 M KOH. Batch fermentations were run for 120 h.

### 2.11 Spore assay

Spore assays were carried out either in flasks for a quick screen or in pH controlled batch cultures (see Section 2.10) to enable efficient sporulation. In flasks, 200 ml CBM-S broth supplemented with 0.25 mM phosphate buffer (pH 7.3, Steiner et al., 2012) were inoculated from CBM-S overnight culture to a final OD_600_ of 0.1 and grown anaerobically for 120 h at 37 °C. After incubation cultures were shaken thoroughly and 100 µl samples taken in duplicate to be treated at 80 °C for 10 min and plated on RCM agar plates to quantify the number of heat resistant colony forming units (CFUs) per ml. pH-controlled (5.5) batch cultures were grown for 120 h. Cells were normalized to the lowest OD_600_ in a final volume of 5 ml PBS buffer, centrifuged (10 min, 10,000*g*, 4 °C), re-suspended in 200 µl PBS buffer and heated to 80 °C for 30 min. Serial dilutions in a total volume of 1 ml PBS were carried out and 10 µl aliquots were spotted onto RCM agar plates. The plates were incubated for 24 h at 37 °C before colonies were enumerated. The sporulation efficiency was determined as number of heat-resistant CFU/ml of culture, that germinate and grow. All results were confirmed microscopically.

### 2.12 HPLC analysis of glycerol, glucose and metabolites

Samples (2 ml) of *C. pasteurianum* cultures from bottle fermentations were collected, centrifuged (10 min, 16,000g, 4 °C) and cell-free supernatants stored at -80 °C until analysed. Cell-free supernatants were thawed on ice, mixed with an equal volume (200 µl) of internal standard solution (80 mM valeric acid [Sigma-Aldrich, Dorset, UK] in 0.005 M H_2_SO_4_), filtered through a 0.22 µm HPLC certified syringe filter (Whatman® Spartan® 13/0.2 RC; GE Healthcare Life Sciences, Little Chalfont, UK) and transferred into a HPLC vial with a 100 µl insert. Substrate (glucose, glycerol) and fermentation products (acetate, acetone, butanol, butyrate, lactate, ethanol, PDO) were analysed by the use of a Dionex UltiMate 3000 HPLC system (Thermo Fisher Scientific, Loughborough, UK) equipped with a Bio-Rad Aminex HPX-87H (Hertfordshire, UK) column, a refractive index (RI) and diode array detector (DAD) at UV 210 nm at an isocratic flow rate of 0.5 ml/min of 0.005 M H_2_SO_4_ as mobile phase and a column temperature of 35 °C for 55 min. The injection volume was 20 µl. If required samples were diluted using reverse osmosis (RO) water. Standard concentrations ranged from 0.98 to 250 mM. For glycerol two additional concentrations of 500 mM and 750 mM were employed. Signal analysis was performed using the Chromeleon 7.2 Chromatography Data System (Thermo Fisher Scientific, Loughborough, UK). Statistical analysis of significance was performed using the PRISM (GraphPad Software, La Jolla, USA) employing Fisher's *t*-test. The significance level (α-value) was set at 0.05.

### 2.13 Identification of putative Rex boxes in *C. pasteurianum*

The *C. pasteurianum* genome was analysed for the presence of Rex binding boxes using the Rex box consensus sequence (5’-TTGTTAANNNNTTAACAA) reported by Ravcheev et al. (2012) with the ’Virtual Footprint’ algorithm (Münch et al., 2005) allowing 2 mismatches in a similar fashion to Wietzke and Bahl (2012). As the *C. pasteurianum* genome was not available in the PRODORIC database (Münch et al., 2003) to which ’Virtual Footprint’ is linked, the results were analysed manually. Each ‘hit’ was searched against the genome of *C. pasteurianum* DSM 525 (Poehlein et al., 2015) using the Artemis genome browser (Rutherford et al., 2000). The information extracted was the locus tag, gene name if applicable, protein function and location of the target sequence in respect of the genome context. Only target regions in intergenic regions with a putatively regulated gene up- or downstream were considered. For every entry in the list the distance to the closest start codon was measured and a frequency plot with a bin size of 10 bp was considered. The result is a positive skewed distribution with the maximal target site occurrence between 80 bp and 90 bp from the start codon which dropped to near zero occurrences after 250 bp from the start codon. This observation led to further filtering the list with a threshold of 250 bp distance to the start codon. The resulting list comprised 40 sequences which were used in an iterative approach to run ’Virtual Footprint’ with a new Position Weight Matrix (PWM). This led to a list of 113 targets which were filtered to exclude hits in coding regions of genes and in intergenic regions with antidromic genes. The final list comprised 47 targets which putatively regulate downstream genes (Supplementary Table S2).

## 3 Results

### 3.1 Improved transformation of *C. pasteurianum* DSM 525

*C. pasteurianum* ATCC 6013 has been shown to be transformable with several pMTL80000 modular vectors (Heap et al., 2009) at efficiencies of up to 7.5×10^4^ transformants μg^-1^ DNA (Pyne et al., 2013). This high frequency was reliant on circumventing the activity of the endogenous *Cpa*AII restriction-modification system by *in vivo* methylation of its recognition site (5’-CGCG-3’) using the M.*Fnu*DII methyltransferase (Pyne et al., 2013). As the *E.coli* host employed (Top10) was *dam+,* the activity of the restriction enzyme CpaII, a *Mbo*I/*Dpn*II-type restriction endonuclease previously identified as *CpaI* (Richards et al., 1988), was of no consequence. Here we used either *in vitro* or *in vivo* methylation by M.SssI or M.BepI, respectively. The former methylates all cytosine residues within the double-stranded recognition site 5’-^m^CG-3’ (Renbaum et al., 1990), whereas similar to M.*Fnu*DII, M.BepI methylates the external cytosine (5’-^m^CGCG-3’) of the *Cpa*AII recognition site (Lunnen et al., 1988). Whilst either methylation procedure allowed transformation of both ATCC 6013 and DSM 525 (equivalent to ATCC 6013) to be obtained with plasmids pMTL83151 (pCB102 replicon) and pMTL85151 (pIM13 replicon) (Heap et al., 2009) using our published method (Cooksley et al., 2012), the success rate and frequencies obtained was very low, 1.5×10^1^ transformants μg^-1^ DNA. After adapting the method published by Pyne et al. (2013) with added sucrose in growth medium and extra step of glycine wash, the transformation frequency was improved to 1.6×10^2^ transformants μg^-1^ DNA, still two orders of magnitude lower than reported by Pyne et al. (2013) of which up to 7.5×10^4^ transformants μg^-1^ DNA was achieved. Plasmids pMTL82151 (pBP1 replicon) and pMTL84151 (pCD6 replicon) could not be transformed.

We hypothesised that one reason for the low number of transformants was that those cells that were competent for the acquisition of plasmid DNA represented rare mutant cells present within the population. This hypothesis was tested by curing the plasmid of a randomly selected transformant of DSM 525 through repeated subculture in the absence of the selective antibiotic, and then retesting the cured strain to see if the plasmid-free strain was more amenable to electroporation. The strain was found to transform at frequencies of 2.6×10^5^ transformants μg^-1^ DNA. Moreover, similar frequencies were observed with plasmids pMTL85151, pMTL82151 and pMTL84151 (Heap et al., 2009). Whole genome sequencing of the strain with higher frequency *C. pasteurianum* DSM 525-H1 (designated CRG4111) and compared to the parental strain *C. pasteurianum* DSM 525 (Poehlein et al., 2015) indicated that two SNPs had arisen within two distinct open reading frames, that of CLPA_c13710 and CLPA_c33080 (Poehlein et al., 2015). Unexpectedly, neither encoded protein was obviously associated with restriction/ modification. Both SNPs resulted in frame-shifts in the encoded sequence. CLPA_c13710 encodes a predicted β-lysine N6-acetyltransferase (*ablB*) which was reduced from 283 amino acids to 176 residues in the hypertransformable mutant. CLPA_c33080 encoded a histidine kinase (ResE9) and leads to a frameshift that reduces the protein from 615 to 160 amino acids in length. To ascertain whether the general phenotypic properties of the CRG4111 mutant strain had been affected by the SNPs, comparative growth experiments were performed on glycerol as the carbon source. The growth and glycerol consumption rates of the mutant were similar to the parental strain. Aside from a small increase in the levels of PDO and a small reduction in *n*-butanol, all other measured metabolites (ethanol, lactate, acetate and butyrate) were essentially the same between the two strains. On this basis, the strain was taken forward for metabolic engineering studies.

### 3.2 Implementation of a gene system roadmap

We have previously described the implementation of allelic exchange, gene KO systems in specifically generated *pyrE* mutants of *Clostridium acetobutylicum* (Ehsaan et al., 2016) and *Clostridium difficile* (Ng et al., 2013), and most recently *Geobacillus thermoglucosidasius* (Sheng et al., 2017). The generation of mutants by allelic exchange is facilitated by the use of replication defective, or pseudo-suicide vectors (Cartman and Minton, 2010), and the incorporation into the vector of a functional copy of a heterologous *pyrE* gene (encoding orotate phosphoribosyl transferase) that serves as a counter/ negative selection marker. This approach serves as a general roadmap for the implementation of gene KO systems in clostridia (Minton et al., 2016). Pivotal are the generation of a *pyrE* truncation mutant using Allele-Coupled Exchange (ACE) technology (Heap et al., 2012) and the identification of an appropriately replication defective (pseudo-suicide) vector.

Segregation stability studies (see Supplementary Information) on transformed cells carrying the various pMTL80000 modular vectors established that plasmids based on the pIM13 replicon were suitably defective, with 99% of the cells losing the plasmid after eight 12 h subcultures (approximately 51 generations) in media lacking antibiotic selection. Accordingly, a pIM13-based vector (pMTL-KS01) was constructed (Fig. 2 and Materials and Methods) equivalent to the ACE plasmids pMTL-YN18 (Ng et al., 2013) and pMTL-JH12 (Heap et al., 2012) and a *pyrE* (CLPA_c26850) truncation mutant generated as previously described (Heap et al., 2012, Ng et al., 2013). In essence, thiamphenicol (Tm) resistant (^R^) transformants in which pMTL-KS01 had integrated into the CRG4111 genome *via* a longer Right Homology Arm (RHA) were identified as larger, faster growing colonies on RCM media supplemented with 15 µg/ml Tm (RCM_Tm15_) and purified by restreaking onto the same selective media. Of the 24 selected colonies, all were shown to be pure single crossover mutants through PCR screening using appropriate primers (see Supplementary Table S1). To select for a subsequent double crossover excision event involving the shorter Left Homology Arm (LHA) and the generation of the required 5-fluoroorotic acid (FOA), resistant *pyrE* deletion mutant, single crossover integrants were restreaked onto CBM media supplemented with 5 µg/ml uracil (Ura) and FOA at the experimentally determined MIC (600 µg/ml) of the parental strain (CBM_Ura5, FOA600_). From a total of eight FOA^R^ clones obtained, subsequent restreaking and testing of phenotype on appropriately supplemented media demonstrated that in addition to being FOA^R^, three were uracil auxotrophs (required supplementation of CBM media with exogenous uracil for growth) and sensitive (^S^) to Tm (could not grow if Tm was present). From PCR screening using primers flanking the *pyrE* gene and the subsequent sequencing of the amplified DNA fragment, two of the three clones were shown to carry the desired modification. This equated to a deletion extending from nt 335–582 of CLPA c26850, and the concomitant insertion of the *lacZα*/MCS originating from pMTL-KS01. One strain was selected for further use and designated CRG4273. To demonstrate that CRG4273 could be restored to wildtype (WT) through correction of the *pyrE* allele an ACE, *pyrE* repair vector was made (pMTL-AGH12) equivalent to pMTL-YN1/2 (Ng et al., 2013) and pMTL-ME6 (Ehsaan et al., 2016) (see Fig. 2). The region of homologous DNA in this plasmid essentially comprises a contiguous region of DNA from the *pyrE* locus that includes a complete copy of the *pyrE* gene and downstream region equivalent to the 1200 bp RHA of plasmid pMTL-KS01. This plasmid was transformed into CRG4273 and single crossover integrants identified as larger, faster growing colonies on CBM_Tm15, Ura5_ agar plates. Growth from several representative colonies were thereafter restreaked onto CBM agar lacking any supplementation, and single colonies patch plated onto RCM and RCM_Tm15_ agar plates to identify those clones in which the excised plasmid had been lost. The *pyrE* locus of three representative clones was amplified by PCR using appropriate PCR primers (KS004_Cpa_pyrE_gen_Fw and KS004_Cpa_pyrE_gen_Rev, Supplementary Table S1) and subjected to Sanger sequencing. This data confirmed that the locus had been restored to WT in all three cases. One was chosen for storage and designated CRG5570 (Table 1).

### 3.3 Exemplification of the knock-out vector pMTL-KS15

The availability of the *pyrE* deletion mutant CRG4273 is a prerequisite for the use of a functional heterologous *pyrE* gene as a counter selection marker in gene knock-out (KO) by allelic exchange. Accordingly, a KO vector broadly equivalent to pMTL-YN3/4 (Ng et al., 2013), pMTL-ME3 (Ehsaan et al., 2016) and was made, pMTL-KS15 (Fig. 2), and exemplified through the in-frame deletion of the master regulator of sporulation, *spo0A*. Precise details of the vector are given in Materials and Methods, but is essentially based on the pIM13 replicon and incorporates the *C. perfringens catP* selectable marker and a heterologous *pyrE* gene derived from *C. acetobutylicum* (Liew et al., 2017), as opposed to the *C. sporogenes pyrE* gene used in the previous studies (Ehsaan et al., 2016, Ng et al., 2013).

The KO cassette for *spo0A* (CLPA c19180) was generated by SOE PCR as described in Materials and Methods and cloned between the *Sbf*I and *Nhe*I sites of pMTL-KS15. Following transformation of the resultant plasmid (pMTL-KS15::spo0A, Table 1) into CRG4273 and plating on RCM_Tm15_ agar plates, putative single crossover integrants were identified as faster growing, larger colonies. Their identity as pure single crossover integrants was confirmed by appropriate PCR (primers KS005_spo0A_genome_Fw, KS005_rex_genome_Rev, Supplementary` Table S1) and then one of the twelve identified grown overnight in CBMS_FOA600, Ura5_ broth to allow the double crossover event to take place. After the overnight incubation the culture was centrifuged, re-suspended and serial dilutions plated to single colonies on RCM agar plates. Selected colonies were patch plated onto RCM_Tm15_ and un-supplemented RCM agar plates and onto CBM media lacking uracil. Growth on RCM alone confirmed loss of the plasmid, and the encoded *catP* and *pyrE* genes, following its excision. PCR screening with flanking primers (KS005_spo0A_genome_Fw and KS005_spo0A_genome_Rev, Supplementary Table S1), was then used to distinguish between those excision events that had generated the desired deletion as opposed to return of the cell to WT. A total of 17 of the 50 putative KO clones screened generated a smaller DNA fragment consistent with the intended deletion in *spo0A* (Supplementary Fig. S1). Through Sanger sequencing of the amplified DNA fragment, the presence of the expected *spo0A* deletion was confirmed in all 17 strains. One of the clones was selected (CRG5514) and restored to uracil prototrophy using the ACE vector pMTL-AGH12 as previously described. The final strain, carrying only the *spo0A* mutation, was designated CRG5516. In parallel, a derivative of pMTL-KS12 was constructed (pMTL-KS12::spo0A*, Table 1) in which a functional copy of the *C. pasteurianum spo0A* gene, together with its native promoter, was inserted into the MCS between the LHA and RHA. Use of this strain to restore the *pyrE* mutation of CRG5514 to WT led to insertion of *spo0A* into the chromosome for complementation studies. The genotype of the strain generated, CRG5518, was confirmed by PCR amplification of the *pyrE* locus and inserted *spo0A* gene and nucleotide sequencing of the DNA fragment amplified.

### 3.4 Phenotypic analysis of *spo0A* deletion mutant

**Fig. 3:**
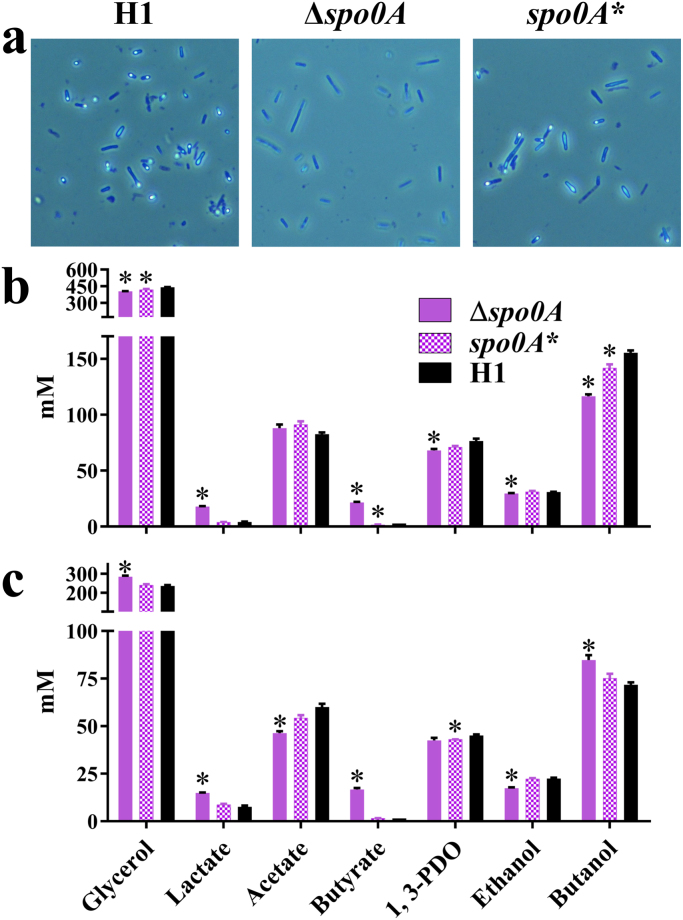
Comparison of sporulation and fermentation phenotypes of *C. pasteurianum*-H1 (H1), *C. pasteurianum*-H1Δ*spo0A* (Δ*spo0A*) and *C. pasteurianum*-H1-*spo0A* complementation (spo0A*). a) Spores can be observed in H1 and spo0A* whereas Δ*spo0A* as expected is incapable of producing spores. b), c) Pure glycerol fermentation in serum bottles with 60 g/l glycerol in Biebl medium (b) and CGM (c) was carried out for 48 h with fermentation being visibly completed after 24 h which is the time point shown for glucose consumption and product formation. * indicate statistical significance in *t*-test α>0.05 of deletion or complementation strain against H1. Error-bars indicate standard error of three replicates.

As expected, after 5 days of growth in a pH-controlled batch cultures (pH 5.5), no heat resistant CFUs were obtained after plating on RCM of the *spo0A* mutant strain CRG5516. In contrast, cultures of the WT (CRG4111) and the complemented mutant strain CRG5518 yielded 1×10^8^ heat resistant CFU/ml. In keeping with these measurements, spores were easily detected using phase contrast microscopy in the cell suspensions of CRG4111 (parent) and CRG5518 (*spo0A,* complemented), whereas none could be detected in cultures of CRG5514 (*spo0A*) (Fig. 3a). To analyse the effects of the *spo0A* deletion on solvent profiles, strains CRG4111 (parent), CRG5516 (*spo0A*) and CRG5518 (*spo0A*, complemented) were grown in 50 ml Biebl medium supplemented with 60 g/l glycerol (Fig. 3b). All strains exhibited a similar growth rate but the pH of the *spo0A* mutant (CRG5516) dropped to a lower level than the parent and complemented strain, presumably due to increased production of lactate and butyrate. Glycerol uptake, acetate production and reassimilation and ethanol production are similar in all strains.

Butanol and PDO levels were slightly lower in the mutant in Biebl medium. In contrast, the complemented *spo0A* mutant strain (CRG5518) produced slightly higher levels of these solvents. The data obtained was at some variance from that of Sandoval et al. (2015) who undertook a similar analysis of a *spo0A* mutant of *C. pasteurianum*. However, these authors used rich media (CGM) as opposed to the minimal media employed here. We, therefore, repeated our analysis using CGM (Fig. 3c). In this medium, the production of lactate and butyrate by the *spo0A* mutant was significantly increased compared to the parent strain, CRG4111. Interestingly, the mutant (CRG5516) produced much more acetate in the initial stages of the fermentation, but then reassimilated the acid from 12 h onwards. No equivalent reassimilation was seen in either the parental (CRG4111) or complemented (CRG5518) cultures. In common with the observations of Sandoval et al. (2015), compared to the parental strain (CRG4111), glycerol consumption was significantly increased together with the production of higher levels of *n-*butanol. In contrast to Sandoval et al. (2015), however, the PDO levels of the *spo0A* mutant (CRG5516) were not reduced compared to the wildtype but remained unaltered.

### 3.5 Construction of deletion mutants in genes involved in solvent production

To investigate whether solvent profiles could be altered in favour of *n-*butanol production we targeted genes encoding the redox-responsive regulator Rex, the main hydrogenase (*hydA*) and the glycerol dehydratase (*dhaBCE*).

Rex has been previously shown to control the expression of *n*-butanol biosynthetic genes in response to the cellular NADH/NAD^+^ ratio in *C. acetobutylicum* where its disruption led to reduced acid production and increased solvents yields (Wietzke and Bahl, 2012). In *C. acetobutylicum* the *rex* gene resides immediately 5’ to the *crt-bcd-etfAB-hbd* operon responsible for butyryl-CoA biosynthesis (Wietzke and Bahl, 2012). An equivalent gene (CLPA_c28640) is found in the same context in *C. pasteurianum* and encodes a 213 amino acid protein exhibiting 76% amino acid sequence identity to the *C. acetobutylicum* Rex. In the case of hydrogenase, a number of different [FeFe]-hydrogenases and [NiFe]-hydrogenase exist in *C. pasteurianum* but little information is available concerning their specific individual roles in hydrogen formation and redox balance. BLAST analysis showed that the protein encoded by CLPA_c00280 exhibits 71% identity to the well described *C. acetobutylicum* hydrogenase, *hydA* (Santangelo et al., 1995). Accordingly, KO cassettes to bring about the deletion of CLPA_c28640 (Rex) and CLPA_c00280 (Hyd) were generated by SOE PCR, cloned into pMTL-KS15 and the resultant mutants generated by allelic exchange using our standard procedure (Table 1, Supplementary Fig. S1 and Materials and Methods).

Simplistically, one way to increase butanol titres when growing on glycerol might be to eliminate PDO production. The conversion of glycerol to PDO involves a two-step transformation by glycerol dehydratase (DhaBCE), encoded by *dhaBCE* (CLPA_c22770-CLPA_c22760-CLPA_c22750) and PDO dehydrogenase (DhaT), encoded by *dhaT* (CLPA_c22740). Given the inability of Pyne et al. (2016) to isolate a clean mutant of *dhaT* in the presence of the toxic intermediate 3-hydroxypropionaldehyde (3-HPA) (Barbirato et al., 1996, Maervoet et al., 2014), we chose to ablate PDO production by eliminating the synthesis of glycerol dehydratase through deletion of the entire *dhaBCE* operon. Accordingly, a KO cassette to achieve this was generated by SOE PCR, cloned into pMTL-KS15 and the resultant KO plasmid used to generate a *dhaBCE* mutant (CRG5532) by allelic exchange using our standard procedure (Table 1, Supplementary Fig. S1 and Materials and Methods).

**Fig. 4:**
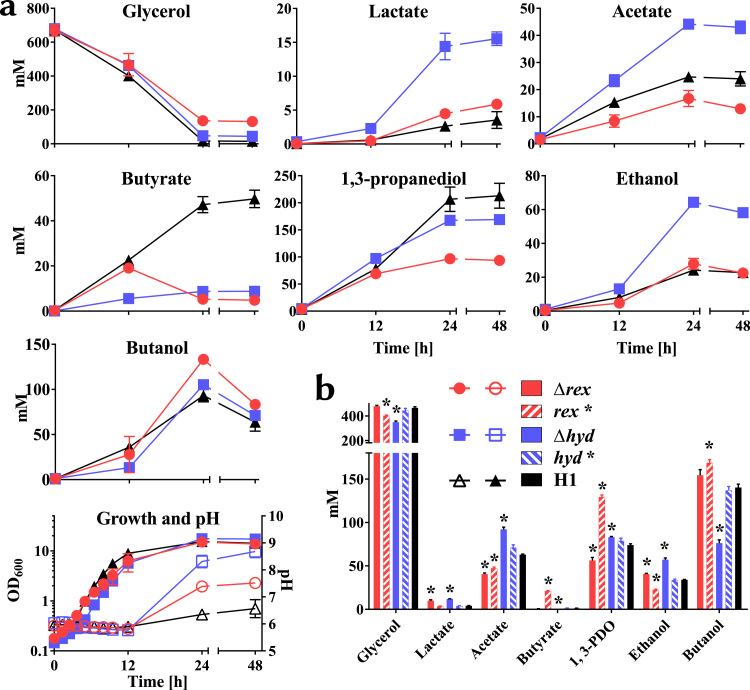
Pure glycerol fermentations of *C. pasteurianum*-H1 (H1), *C. pasteurianum*-H1Δ*rex* (Δ*rex*), *C. pasteurianum*-H1-*rex* complementation (rex*), *C. pasteurianum*-H1Δ*hyd* (Δ*hyd*) and *C. pasteurianum*-H1-*hyd* complementation (hyd*). a) Bioreactor fermentation with 60 g/l glycerol in Biebl medium with 1 g/l yeast extract at pH 6 was carried out for 48 h with fermentation being visibly completed after 24 h. Error-bars indicate range of two fermentations. b) Histogram showing product formation of serum bottle fermentation of deletion strains and complementations. Glycerol usage and product formation is shown after 24 h. * indicate statistical significance in a *t*-test α>0.05 of deletion or complementation strain against H1. Error-bars indicate standard error of three replicates.

**Fig. 5:**
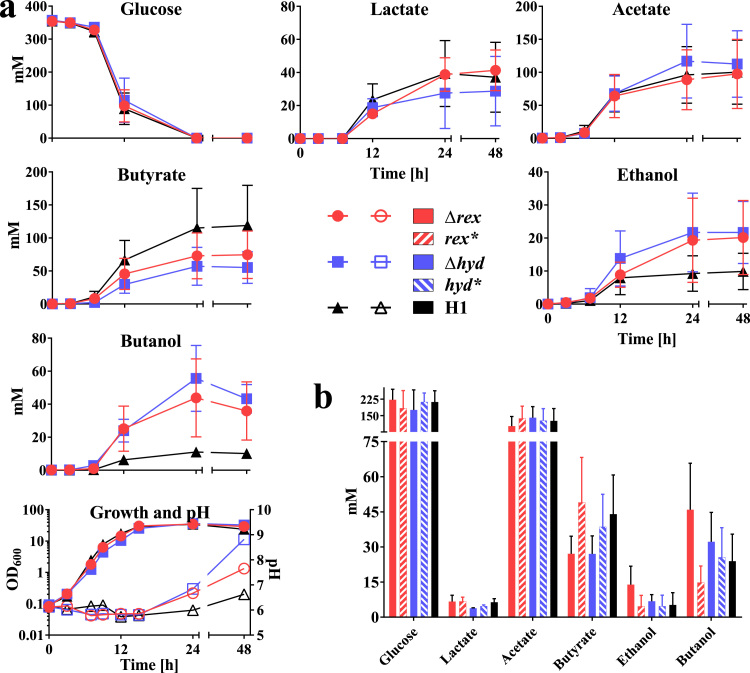
Glucose fermentations of *C. pasteurianum*-H1 (H1), *C. pasteurianum*-H1Δ*rex* (Δ*rex*), *C. pasteurianum*-H1-*rex* complementation (rex*), *C. pasteurianum*-H1Δ*hyd* (Δ*hyd*) and *C. pasteurianum*-H1-*hyd* complementation (hyd*). a) Bioreactor fermentation with 60 g/l glucose in Biebl medium at pH 6 was carried out for 48 h with fermentation being visibly completed after 24 h. Error-bars indicate range of two fermentations. b) Histogram showing product formation of serum bottle fermentation of deletion strains and complementations. Glucose usage and product formation is shown after 24 h. Error-bars indicate standard error of three replicates.

**Fig. 6:**
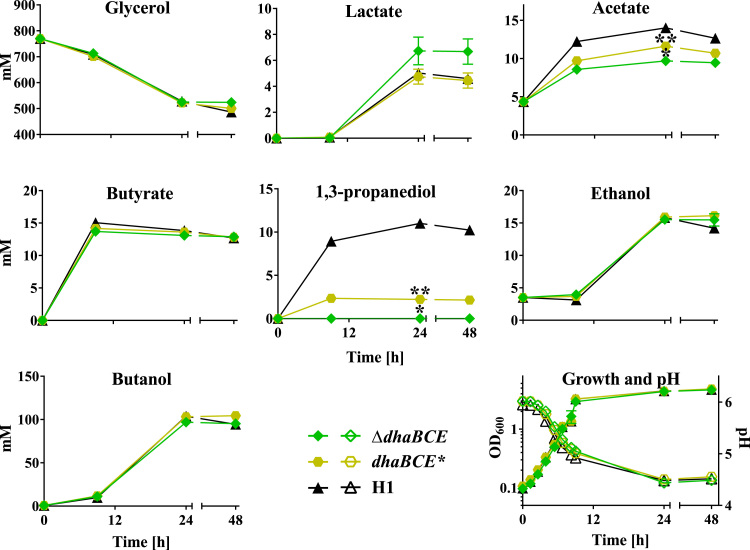
Pure glycerol fermentations of *C. pasteurianum-H1* (H1), *C. pasteurianum*-H1Δ*dhaBCE* (Δ*dhaBCE*) and *C. pasteurianum*-H1-*dhaBCE* complementation (dhaBCE*). Serum bottle fermentation with 60 g/l glycerol in 2x YT medium was carried out for 48 h with fermentation being visibly completed after 24 h. * indicates statistical significance of Δ*dhaBCE* and ** of dhaBCE* in a *t*-test α>0.05 against H1. Error-bars indicate standard error of three replicates.

In all three cases (*rex*, *hyd* and *dhaBCE*), the mutants were restored to uracil prototrophy, through ACE-mediated correction of the *pyrE* mutation, and complemented through the integration of a functional copy of the deleted gene with the native promoter concomitant with restoration of the mutant *pyrE* allele to WT (Materials and Methods). Phenotypic analysis of all mutants, and their complemented equivalents, involved the determination of carbon utilisation as well as the concentration of the following fermentation products: acetate, butyrate, lactate, acetone, ethanol, PDO and *n*-butanol. All studies, with the exception of the glycerol fermentation of the *dhaBCE* mutant (CRG5534), were carried out in batch culture in bioreactors using Biebl medium supplemented with 1 g/l yeast extract and 6% (w/v) glycerol or with glucose omitting yeast extract. Fermentation profiles are shown in Fig. 4, Fig. 5, Fig. 6.

### 3.6 Phenotypic analysis of the *rex* and *hyd* mutants

In glycerol media (Fig. 4), both mutants grew at broadly equivalent growth rates compared to the WT, but achieved higher final pH values, being 8.7±0.2 and 7.5±0.1 after 48 h for the *hydA* and *rex* mutant, respectively compared to 6.6±0.3 in the WT culture. Almost complete glycerol consumption took place in the WT and *hydA* culture (2% and 7% remaining, respectively), with the rex mutant culture containing 20% after 48 h. The *hydA* mutant produced the highest titres of both lactate and acetate, but significantly reduced amounts of butyrate. A slightly greater reduction in butyrate production was observed with the *rex* mutant compared to the *hydA* mutant. In terms of lactate and acetate production, the former were slightly increased compared to the WT, whereas the latter marginally decreased. In the case of solvents, PDO production was reduced in both mutants, with the largest reduction occurring in the *rex* mutant, which decreased by 53% compared to the WT, with only a 19% reduction being evident in the *hydA* mutant. Ethanol formation in the *hydA* mutant was significantly elevated (64.3±3.2 mM after 24 h) over both the WT and *rex* mutant which, with respective titres of 24.2±1.4 mM and 28.0±3.2 mM, were broadly equivalent. Butanol formation was highest in the *rex* mutant, with the titres achieved after 24 h being 133.3±1.8 mM compared to 105.1±0.0 mM in the *hydA* mutant and 93.2±5 mM in the WT. After 24 h the ethanol and butanol levels decreased, most likely due to solvent extraction by gas stripping from the nitrogen bubbling through the reactor.

On glucose (Fig. 5), the growth rate and glucose utilisation rates were little affected in either mutant compared to the WT. Differences in acid production were also less marked in the two mutants compared to when grown on glycerol. Acetate production in the *hydA* mutant was slightly increased compared to the WT and *rex* mutant, which were essentially the same. On the other hand, lactate production in the *hydA* mutant was only slightly reduced compared to the *rex* mutant and WT. Butyrate produced was decreased in both mutants compared to the WT, with the larger reduction being seen in the *hydA* mutant, producing 57.0±14.3 mM compared to 115.0±30.0 mM in WT culture after 24 h. Ethanol and *n*-butanol titres, and in particular the latter solvent, were increased in both mutants, with *hydA* demonstrating a 2.3-fold and 5-fold higher final titre than the WT, respectively, at 24 h.

Whilst the fermentation profile of the complemented *hydA* mutant was essentially restored to that of the WT, complementation of the *rex* mutant was less clear cut. Thus, whereas the PDO levels of the mutant were reduced in comparison to the WT and the *n*-butanol levels increased, the complemented strain produced higher amounts of PDO and reduced titres of *n*-butanol, relative to the WT. Such a phenotype would be indicative of higher than normal expression of *rex* in the complemented strain, however, the *rex* gene was inserted at *pyrE* under the control of its native promoter. In certain instances expression of genes can be affected by the proximity of the gene to the origin of chromosome replication (Sauer et al., 2016). This does not appear to be the case here, as the distance of *rex* and *pyrE* to the origin are relatively similar with *rex* being slightly further away from the origin. In *C. kluyveri*, *rex* is self-regulated through the agency of a Rex box, repressor site immediately upstream of the structural gene (Hu et al., 2016). Whilst such an operator site was not evident in the *C. pasteurianum rex* promoter region used to express the complementing copy of *rex*, a Rex box was found 340 bp upstream of the *rex* start codon within the upstream CLPA_c28650 gene (genome position 3068768- 3068786). As the promoter region used in the complemented strain lacks this sequence it is likely that *rex* expression is deregulated, resulting in the observed produced higher amounts of PDO and reduced titres of *n-*butanol, relative to the WT.

### 3.7 Phenotypic analysis of the *dhaBCE* mutant

Growth of *C. pasteurianum* on glycerol is not redox balanced because cell biomass (_D, 4.3) is more oxidised than glycerol (_D, 4.67). The requirement to oxidise the excess of reducing equivalents generated is met by the PDO pathway which ensures the net oxidation of 1 mol of NADH per 1 mol of PDO formed. The central role of this pathway in maintaining redox balance suggests that the imbalance caused by its inactivation in *C. pasteurianum* would lead to an inability to ferment glycerol. Indeed, the mutant (CRG5534) was unable to grow in a bioreactor in Biebl medium when the carbon source was 6% (w/v) glycerol. The mutant was, however, able to grow in 2x YT containing glycerol (6%, w/v) at equivalent rates to the WT (Fig. 6). The most notable change in fermentation profile was the production of PDO, which, as expected, was eliminated in the mutant (Fig. 6). In contrast, the production of metabolites derived from acetyl-CoA (butyrate, ethanol and *n*-butanol) were largely unaffected, apart from a decrease of acetate production to 9.7 mM at time point 24 h compared to 14 mM in the WT, and an increase in lactic acid production (6.7 mM at time point 48 h compared to 4.6 mM in the WT). The solvent profiles of CRG5534 on glucose where as expected essentially the same as the WT (Fig. S2). In the complemented strain, in which *dhaBCE* has been integrated at *pyrE* together with its native promoter*,* production of PDO was re-instated, although at a lower level than the WT. The lactate and acetate profiles were very similar to that of the WT (Fig. S2).

## 4 Discussion

The use of *pyrE* alleles for gene knock-out forms part of a general ‘roadmap’ for manipulating bacterial genomes (for a review see Minton et al., 2016), the implementation of which is reliant on effective gene transfer into the target organism. In the case of *C. difficile* (Ng et al., 2013) and *G. thermoglucosidasius* (Sheng et al., 2017) no special measures were required to achieve this, whereas in *C. acetobutylicum* the rational circumvention of host restriction barriers proved to be necessary by appropriate methylation of the plasmid DNA being transferred (Ehsaan et al., 2016). Here we showed that an alternative approach is possible through the isolation of a highly transformable variant of the *C. pasteurianum* strain DSM 525, strain CRG4111.

The reason for the increased competence of CRG4111 is unclear. Neither of the two SNPs present are in genes with any obvious association with restriction/ modification. Both are destructive in nature resulting in frame-shifts and premature termination of the encoded proteins. Whether one or the other, or both, are responsible for the phenotype is not immediately apparent. One gene encodes β-lysine N6-acetyltransferase (*ablB*), which, being involved in lysine degradation seems to have no connection with improved DNA transfer. CLPA_c33080 encodes a histidine kinase (ResE9), but does not appear to be part of a two-component system, as no gene encoding a cognate response regulator was located in the immediate vicinity. Interestingly, however, the gene (CLPA_c33090) immediately adjacent to the ResE9 encodes a homologue of an *agr* quorum sensing AgrB protein, responsible for processing and secretion of the AgrD signal molecule. Indeed, an unannotated ORF is present immediately downstream of CLPA_c33090 encoding a 57 amino acid peptide that shares significant identity with clostridial AgrD signal peptides, including 58% with those of *Clostridium tetanomorphum* DSM 665 (KAJ50110.1 and KAJ51453.1), *Clostridium scatologenes* (AKA70070.1) and *Clostridium carboxidivorans* P7 (EET84565.1) and 52% identity to homologues in *Clostridium botulinum* (WP_061325181) and *Clostridium sporogenes* (WP_053818607.1). All of these clostridial species, and many more besides, share the same structural arrangement of genes, corresponding to *agrB*>*agrD*>*resE*. This gene arrangement, was first noted in *C. botulinum*, where it was shown to be involved in the regulation of sporulation and neurotoxin production (Cooksley et al., 2010). The possibility exists that in *C. pasteurianum* at least, this quorum sensing system also regulates a process that can affect the efficiency of DNA uptake.

Regardless of the mechanism responsible for increased competence, our findings have provided a further approach for maximising DNA transfer rates which was used in combination with the developed *pyrE-*based KO system to make a number of mutants (*spo0A, hydA, rex* and *dhaBCE*) that influenced solvent production. A general workflow and schematic for mutant generation is shown in Supplementary Fig. S3. Significantly we were able, for the first time, to completely eliminate PDO production in *C. pasteurianum*. The formation of PDO from glycerol is mediated by the sequential action of glycerol dehydratase (DhaBCE) and PDO dehydrogenase (DhaT). Here we chose to entirely delete the *dhaBCE* region, and as a consequence generated a strain which no longer produced PDO under all conditions tested. Previous attempts to ablate the production of either glycerol dehydratase or PDO dehydrogenase through the use of ClosTron technology met with little success (Pyne et al., 2013). Intron mutants of *dhaB, dhaC* and *dhaE* could not be isolated, whilst an intron insertion in *dhaT* was obtained but the intron delivery plasmid and its encoded LtrA protein could not be cured. Such a phenomenon can occur if the intron insertion is in the sense strand of an essential gene, and LtrA-mediated splicing of the transcribed mRNA provides a route for some production of the encoded protein. However, the intron insertion in the *dhaT* gene was in the antisense orientation. This led to the search for, and apparent discovery of an additional ecotopic, sense strand, intron insertion in *speA* (encoding arginine decarboxylase) which was hypothesised as being essential. One consequence of these difficulties was that Pyne et al. (2016) were unable to eliminate PDO production.

Glycerol is metabolized both oxidatively and reductively in *Clostridium* (Malaviya et al., 2012). The pyruvate-generating oxidative pathway leads to the production of CO_2_, H_2_, and electrons in the form of the reducing equivalent NADH, which is also released during biomass formation. Glycerol, being a highly reduced substrate, results in twice the amount of reducing equivalents compared to using glucose as fermentation substrate. These electrons require acceptors for redox homeostasis, which is precisely the purpose of glycerol reducing pathway producing the highly reduced product PDO. By deleting *dhaBCE* in *C. pasteurianum*, the pathway for funnelling extra reducing power during glycerol fermentation is absent, resulting in the poor growth we observed in the mutant in defined medium. The mutant was, however, able to grow in a rich medium containing yeast extract. The latter ingredient supports growth of the mutant by both providing an alternative source of carbon and acting as a reducing agent. Re-introducing *dhaBCE* back into the deletion mutant reversed the phenotype, confirming the production of PDO in *C. pasteurianum* is important for biomass production and redox balance. Our findings support the hypothesis of Johnson and Rehmann (2016), who found that unlike *C. acetobutylicum*, *C. pasteurianum* glycerol fermentation does not display strong biphasic behaviour, suggesting that PDO production is regulated and further that its regulation may serve to function in redox homeostasis and allow *C. pasteurianum* to behave in a non-biphasic manner.

The Rex response regulator directly senses changes in the redox status of the cell, having a much higher affinity for NADH than for the oxidised nucleotide NAD^+^ (Wang et al., 2008). Under oxic conditions *(i.e.*, when the NADH/NAD^+^ ratio is low) it binds to its target sites to inhibit gene transcription but dissociates from these sites when the NADH/NAD^+^ ratio increases (Brekasis and Paget, 2003). Thus, it was expected that inactivation of Rex would lead to increased levels of those enzymes whose genes were under Rex-mediated control, leading to a consequent change in metabolic profiles. To predict such regulated genes we used the procedure outlined in Materials and Methods to search for potential Rex target sites in the genome. We identified a list of 47 putative Rex box sequences which potentially regulate the adjacent downstream genes (Supplementary Table S2) and defined a consensus sequence for *C. pasteurianum, 5'-* TTGTTAAWNNNTTAACAA. Of the non-fermentative genes previously reported, none were found using our approach (Ravcheev et al., 2012, Zhang et al., 2014). However, other notable putative Rex targets found include CLPA_c05450, a nitroreductase related to *narAB* or *narK* in *C. carboxidivorans* (Zhang et al., 2014), CLPA_c09900, a nicotinamidase family protein, CLPA_c17480 (*spoVD2*) encoding a stage V sporulation protein and CLPA_c39090, a NADPH dehydrogenase.

Given that a high ratio of NADH/NAD^+^ inhibits binding of Rex to DNA, it is expected that Rex-binding sites should be located upstream of genes encoding NADH-dependent enzymes. This is true for the alcohol dehydrogenases putatively involved in butanol and ethanol production (*adhE2* and its two alleles *adhE1* and *adhE4*), indirectly the thiolases that channel carbon into the reductive part of the fermentative pathway (*thlA1* and *thlA2*), the *crt-bcd-etfBA-hbd* operon (crotonase-butyryl-CoA dehydrogenase-electron transfer flavoprotein B/A-hydroxybutyryl-CoA dehydrogenase) which uses 2 NADH to reduce acetyl-CoA to butyryl-CoA, and the reductive part of the *dha* (glycerol dehydratase) operon. In keeping with these observations, production of ethanol and *n*-butanol was elevated in the *rex* mutant when grown on either glycerol or glucose, as was demonstrated in *C. acetobutylicum* (Wietzke and Bahl, 2012).

By the same reasoning, and based on observations made in *C. acetobutylicum* (Ravcheev et al., 2012), we would have expected to find Rex sequences upstream of *ldh* (lactate dehydrogenase), *hydA* (hydrogenase) and *ptb-buk* (phosphotransbutyrylase-butyrate kinase), as was found in other species (Wietzke and Bahl, 2012, Hu et al., 2016), but none were found, even with relaxed algorithm parameters. Nevertheless, contrary to *in silico* expectations and the observations made in *C. acetobutylicum*, the *C. pasteurianum rex* mutant produced more lactate in glycerol fermentation compared to WT (Fig. 4). On the other hand, as expected, butyrate production was significantly reduced in the absence of Rex, and a clear butyrate reassimilation in glycerol fermentation was observed in the *rex* deletion mutant. Butyrate assimilation has been shown in *C. pasteurianum* when feeding external butyrate which led to increased butanol titres (Kao et al., 2013, Lin et al., 2015, Sabra et al., 2014) but reassimilation of butyrate in a standard fermentation was to our knowledge never observed before. Some of the higher *n*-butanol titres observed in the mutant strain could be explained by reassimilation and carbon recycling of butyrate.

Acetone formation was never observed in fermentations using the *C. pasteurianum* WT, in agreement with Biebl (2001), Sandoval et al. (2015) and Pyne et al. (2016). However, in the *rex* deletion mutant butyrate is visibly reassimilated which in the pathway proposed by Malaviya et al. (2012) is coupled to acetone formation *via* the acetoacetyl- CoA: butyrate:CoA transferase in a similar fashion to *C. acetobutylicum* (Jones and Woods, 1986). These findings suggest that *C. pasteurianum* may be able to reassimilate butyrate without the need of acetone production either through the reverse reaction of the butyrate kinase (*buk*) and the phosphotransbutyrylase (*ptb*), as previously suggested (Hüsemann and Papoutsakis, 1989, Millat et al., 2014), or *via* a third, as yet undescribed, mechanism proposed by Millat et al. (2014). Reassimilation of butyrate without acetone production has been described in several *C. acetobutylicum* inactivational mutants, most notably mutants of *ctfA* (Millat et al., 2014), *ptb* (phosphotransbutrylase) and *adc* (acetoacetate decarboxylase) (Lehmann et al., 2012) and butyrate kinase (*buk*) gene (Desai et al., 1999). The fact that *C. pasteurianum* does not produce acetone under any condition tested here, or elsewhere (Biebl, 2001, Pyne et al., 2016, Sandoval et al., 2015), makes this organism an ideal target for investigations of the butyrate and acetate uptake mechanisms not reliant on CtfAB.

Since alcohol production competes for electrons with the formation of molecular hydrogen, reducing the flux of electrons towards H_2_ synthesis might be expected to increase *n*-butanol titres. Various approaches have been reported that were designed to inhibit *in vivo* hydrogenase activity, for example, by the application of artificial electron carriers or sparging the culture with carbon monoxide (Datta and Zeikus, 1985, Hüsemann and Papoutsakis, 1989, Kim et al., 1984, Peguin et al., 1994;). The resultant increased NAD(P)H availability, led to more *n*-butanol and ethanol and less acetone being produced and a consequently improved alcohol: acetone ratio in *C. acetobutylicum*. However, previous attempts to inactivate the gene (*hydA*) encoding the major hydrogenase of *C. acetobutylicum* using the ClosTron were unsuccessful (Cooksley et al., 2012), suggesting that the gene is essential under the conditions tested. Whilst the *C. pasteurianum hydA* gene (CLPA_ c00280) sharing the closest similarity to the *C. acetobutylicum hydA* gene was recently knocked-down by Pyne et al. (2015) using an anti-sense approach, here we were able to generate a null mutant through the deletion of the entire CLPA_c00280 encoding gene.

In keeping with predictions, deletion of *hydA* led to increases in the products of NADH-consuming pathways, with the mutant producing 2.7-fold higher levels of ethanol and 5-fold higher levels of lactate (Fig. 4). In contrast, only a slight increase in *n*-butanol was observed with the mutant generating only 1.1-fold higher levels than the WT. Similar results were obtained with the knock-down mutant of Pyne et al. (2015). Although not a NADH-consuming pathway, a significant increase (1.8-fold) in acetate levels was also seen. Both the acetate and butyrate pathways generate ATP *via* substrate level phosphorylation. One consequence of which was an increase in biomass attained by the mutant (the mutant OD_600_ was 114% higher than the WT) and was characterised by a shorter fermentation time as adjudged by the earlier onset of increase in pH (after 18.7 h as opposed to 22.0 h in the WT). The same observation was made by Pyne et al. (2015). This increase in acetate production was not evident in the study of Dabrock et al. (1992), who used CO to inhibit hydrogenase activity on glucose grown *C. pasteurianum.* Here acetate production was reduced by almost 50%. In this instance, however, all hydrogenase activity would have been affected. The mutant strain utilised in our study, and that of Pyne et al. (2015), is only affected in the production of HydA (CLPA_c00280). Strain *C. pasteurianum* DSM 525 contains 3 more genes/ enzymes (CLPA_c07060-70, CLPA_c33960 and CLPA_c37830) (Poehlein et al., 2015) with homology to hydrogenase enzymes. With CLPA_c07060 and CLPA_c07070 *C. pasteurianum* seems to possess a rare [NiFe]-hydrogenase with the genes encoding the small and large subunit, respectively. The other gene encodes an iron only hydrogenase. All of these proteins might compensate for the loss of HydA (CLPA_ c00280).

Unexpectedly, PDO production was significantly reduced in the *hydA* mutant, by 19% compared to the WT (Fig. 4). This is counter intuitive as the PDO pathway is NADH-consuming. The study of Pyne et al. (2016) also reported such a reduction in levels of PDO (in this case 30%) in their knock-down mutant, while noting that PDO levels can vary widely between fermentations – an observation also made in other studies (Biebl, 2001, Taconi et al., 2009). Our data reinforces the view that the reduction in PDO production that results from ablation of *hydA* function is a real phenomenon. It should also be noted that the reduction in PDO and accompanying increase in biomass is contrary to suggestion of Biebl (2001) that the production of reducing equivalents by biomass build up has to be equalled by production of PDO to regenerate the pool of NAD^+^ to NADH.

## 5 Conclusion

In the current report we have developed and exemplified a robust tool kit for the metabolic engineering of *C. pasteurianum* based on the use of *pyrE* alleles. To maximise gene transfer we deployed a novel approach based on the isolation of a highly transformable variant within the host population. In keeping with previous work on other Gram-positive chassis, we once again showed the advantage of using a *pyrE* minus strain as the host through the rapid complementation of the deletion mutants by the integration of the complementing gene into the genome at the *pyrE* locus concomitant with ACE-mediated restoration of prototrophy. The system was used to make in-frame deletion mutants of pivotal genes involved in solvent production, namely *hydA* (hydrogenase), *rex* (Rex response regulator) and *dhaBCE* (glycerol dehydratase). We were, for the first time in *C. pasteurianum*, able to eliminate PDO synthesis and demonstrate its production was essential for growth on glycerol as a sole carbon source. Inactivation of both *rex* and *hydA* resulted in increase in *n-*butanol titres, representing the first steps towards improving the utilisation of *C. pasteurianum* as a chassis for the industrial production of this important chemical.

## Competing interests

All of the authors declare that they have no competing interests.

## Figures and Tables

**Table 1:** Strains and plasmids used in this study. *C. pa*. = *C. pasteurianum*.

Name	Designation	Properties	Source
*C. pa.* ATCC 6013	CRG4080	wild type, type strain	ATCC
*C. pa.* DSM 525	CRG4091	wild type, type strain	DSMZ
*C. pa.* DSM 525-H1	CRG4111	hypertransformable strain based on DSM 525	This study
*C. pa.* DSM 525-H1Δ*pyrE*	CRG4273	ACE *pyrE* truncation mutant	This study
*C. pa.* DSM 525-H1Δ*pyrE*Δ*spo0A*	CRG5514	ACE *spo0A* deletion mutant with Δ*pyrE* background	This study
*C. pa*. DSM 525-H1Δ*spo0A*	CRG5516	ACE *spo0A* deletion mutant with *pyrE* repaired	This study
*C. pa.* DSM 525-H1::spo0A*	CRG5518	ACE *spo0A* complementation in *pyrE* locus	This study
*C. pa.* DSM 525-H1Δ*pyrE*Δ*rex*	CRG5520	ACE *rex* deletion mutant with Δ*pyrE* background	This study
*C. pa.* DSM 525-H1Δ*rex*	CRG5522	ACE *rex* deletion mutant with *pyrE* repaired	This study
*C. pa.* DSM 525-H1::rex*	CRG5524	ACE *rex* complementation in *pyrE* locus	This study
*C. pa.* DSM 525-H1Δ*pyrE*Δ*hydA*	CRG5526	ACE *hyd* deletion mutant with Δ*pyrE* background	This study
*C. pa.* DSM 525-H1Δ*hydA*	CRG5528	ACE *hyd* deletion mutant with *pyrE* repaired	This study
*C. pa.* DSM 525-H1::hydA*	CRG5530	ACE *hyd* complementation in *pyrE* locus	This study
*C. pa.* DSM 525-H1Δ*pyrE*Δ*dhaBCE*	CRG5532	ACE *dhaBCE* deletion mutant with Δ*pyrE* background	This study
*C. pa.* DSM 525-H1Δ*dhaBCE*	CRG5534	ACE *dhaBCE* deletion mutant with *pyrE* repaired	This study
*C. pa.* DSM 525-H1::dhaBCE*	CRG5536	ACE *dhaBCE* complementation in *pyrE* locus	This study
*E. coli* Top10 x CR1	CRG3131	Strain harbouring plasmid CR1 With M.BepI methylase	This study
pMTL85151		*E. coli*-*Clostridium* shuttle vector (pIM13, *catP*, ColE1 *traJ*, *lacZα* ORF/MCS, T_*Cpa fdx*_)	Heap et al. (2009)
pMTL-AMH101		*catP-pyrE* module used for pMTL-KS15	Liew et al. (2017)
pMTL-KS01		pMTL85151, 300-bp internal *pyrE* fragment, 1200-bp fragment immediately downstream of *pyrE*	This study
pMTL-KS03		pMTL85151, 300-bp internal *pyrE* fragment, 1200-bp fragment immediately downstream of *pyrE*, with T_*Ctet gluRS*_	This study
pMTL-KS04		pMTL85151, 937-bp fragment immediately downstream of *pyrE,* 300-bp internal *pyrE* fragment, T_*Ctet gluRS*_, T_*Cpa fdx*_	This study
pMTL-KS05		pMTL85151, 548-bp internal *pyrE* fragment, 1200-bp fragment immediately downstream of *pyrE*	This study
pMTL-KS08		pMTL85151, 937-bp fragment immediately downstream of *pyrE,* 548-bp internal *pyrE* fragment, T_*Ctet gluRS*_, T_*Cpa fdx*_	This study
pMTL-KS10		pMTL-KS01 features, T_*Ctet gluRS*_, T_*Cpa fdx,*_*Asi*SI	This study
pMTL-KS12		pMTL85151, 548-bp internal *pyrE* fragment, 937-bp fragment immediately downstream of *pyrE*, T_*Ctet gluRS*_, T_*Cpa fdx,*_*Asi*SI	This study
pMTL-KS15		pMTL85151, *pyrE* (*C. acetobutylicum*), T_*Ctet gluRS*_, T_*Cpa fdx*_, 300-bp SHA *pyrE*, *lacZα* ORF/MCS	This study
pMTL-KS16		pMTL85151, *pyrE* (*C. acetobutylicum*), T_*Ctet gluRS*_, T_*Cpa fdx*_, *Asi*SI, 300-bp SHA *pyrE*, *lacZα* ORF/MCS	This study
pMTL-AGH12		1748-bp fragment comprising *pyrE* (35–582 nt) and 1200-bp immediately downstream of *pyrE* cloned into *SbfI*/*Asc*I recognition sites of pMTL-KS12	This study
pMTL-KS12::*spo0A*		1095-bp fragment comprising the 267-bp sequence upstream of *spo0A* and the *spo0A* gene cloned into the *NotI*/*NheI* recognition sites of pMTL-KS12	This study
pMTL-KS12::*rex*		816-bp fragment comprising the 183-bp sequence upstream of *rex* and the *rex* gene cloned into the *NotI*/*NheI* recognition sites of pMTL-KS12	This study
pMTL-KS12::*hyd*		2118-bp fragment comprising the 393-bp sequence upstream of *hyd* and the *hyd* gene cloned into the *NotI*/*NheI* recognition sites of pMTL-KS12	This study
pMTL-KS12::*dhaBCE*		2984-bp fragment comprising the 294-bp sequence upstream of *dhaB* and the *dhaBCE* genes cloned into the *Not*I/*Nhe*I recognition sites of pMTL-KS12	This study
pMTL-KS15::KO_*spo0A*		1400-bp KO out cassette for *spo0A*	This study
pMTL-KS15::KO_*rex*		1364-bp KO out cassette for *rex*	This study
pMTL-KS16::KO_*hyd*		2002-bp KO out cassette for *hyd*	This study
pMTL-KS15::KO_*dhaBCE*		1602-bp KO out cassette for *dhaBCE*	This study

**Table 2:** Solvent and acid yields of *C. pasteurianum* DSM 525 (WT) and its various mutant derivatives when grown in bioreactors on either glucose or glycerol as the carbon source. Mutants were: *C. pasteurianum* DSM 525-H1Δ*rex* (Δ*rex*, CRG5522); *C. pasteurianum* DSM 525-H1Δ*hyd* (Δ*hyd*, CRG5528), and; *C. pasteurianum* DSM 525-H1Δ*dhaBCE* (Δ*dhaBCE*, CRG5534). Abbreviations used: butanol (BuOH), ethanol (EtOH), 1,3-propanediol (PDO), solvents (EtOH, BuOH, PDO), acids (acetate, butyrate, lactate). Carbon recovery was calculated by assuming 3.5 g/l dry-weight per 10 OD values (Sarchami et al., 2016), carbon dioxide desorption as described by Percheron et al. (1995) and the assumption that 46.2% of dry-weight is carbon (Papoutsakis and Meyer, 1985). The carbon fraction of yeast extract was neglected. *Fermentation of Δ*dhaBCE* was undertaken in serum bottles along with a wild type control.

Carbon source		60 g/l glycerol					60 g/l glucose			
Medium		Biebl plus 1 g/l yeast extract			2x YT*		Biebl			
Strain		Δ*rex*	Δ*hyd*	WT	Δ*dhaBCE*	WT	Δ*rex*	Δ*hyd*	Δ*dhaBCE*	WT
Growth characteristics										
	Specific growth rate [h−1]	0.32±0.04	0.35±0.01	0.42±0.01	0.39±0.00	0.40±0.00	0.15±0.02	0.11±0.01	0.13±0.03	0.16±0.03
	Doubling time [min]	135±15	119±3	100±1	106±1	105±0	90±9	103±10	85±4	81±12
	Max. OD	15.0±0.5	17.5±0.6	15.3±0.3	4.38±0.12	4.48±0.01	34.9±1.0	36.2±0.1	34.8±2.5	34.3±2.7
	Carbon recovery [%]	88.9±2.7	91.4±1.3	90.5±4.4	114.4±6.0	127.8±5.4	70.7±14.3	83.5±15.0	72.3±13.9	73.3±14.9

Selectivity [M/M]										
	BuOH/Solvents	0.516±0.002	0.312±0.003	0.289±0.024	0.862±0.002	0.795±0.001	0.703±0.015	0.729±0.021	0.635±0.027	0.563±0.051

Yield [M/M]										
	BuOH/C-Source	0.250±0.005	0.166±0.004	0.142±0.003	0.399±0.019	0.431±0.021	0.124±0.034	0.156±0.029	0.041±0.008	0.031±0.003
	EtOH/C-Source	0.053±0.006	0.102±0.001	0.037±0.000	0.064±0.002	0.066±0.003	0.055±0.018	0.061±0.017	0.025±0.007	0.026±0.008
	PDO/C-Source	0.182±0.000	0.265±0.005	0.317±0.034	0.000±0.000	0.046±0.002	n.a.	n.a.	n.a.	n.a.
	Solvents/C-Source	0.484±0.011	0.533±0.007	0.496±0.030	0.463±0.021	0.542±0.026	0.179±0.052	0.217±0.046	0.066±0.016	0.057±0.001
	Acids/C-Source	0.050±0.006	0.106±0.000	0.114±0.008	0.122±0.009	0.136±0.004	0.567±0.129	0.567±0.153	0.686±0.156	0.708±0.177

## Footnotes

Appendix A Supplementary data associated with this article can be found in the online version at doi:10.1016/j.ymben.2017.01.009.
