# Supplementary material for: Towards improved butanol production through targeted genetic modification of Clostridium pasteurianum
Source: Metab Eng. 2017 Mar;40:124–37. doi: 10.1016/j.ymben.2017.01.009 (PMC5367854; doi:10.1016/j.ymben.2017.01.009)
Supplement: Supplementary Table S1 — Supplementary material [file mmc1.pdf]

SUPPLEMENTARY INFORMATION

Towards improved butanol production through targeted genetic modification of *Clostridium pasteurianum*

Katrin M. Schwarz[a1], Alexander Grosse-Honebrink[a1], Kamila Derecka[a], Carlo Rotta[a], Ying Zhang[a], Nigel P. Minton[a2]

[a]*Clostridia Research Group, BBSRC/EPSRC Synthetic Biology Research Centre (SBRC), University of Nottingham, University Park, Nottingham NG7 2RD, UK.*

[1] these authors contributed equally to the work
[2]corresponding author: mrznpm@exmail.nottingham.ac.uk

Materials and Methods

Electroporation of *C. pasteurianum*

Briefly, 20 ml of 2x YTG broth were inoculated with cells from a well grown overnight culture (OD_{600} of 0.8 to 1.2) to a final OD_{600} of 0.05 and incubated for 3 to 4 h until an OD_{600} of 0.6 to 0.8 was reached. Cells were centrifuged (10 min, 8,500 x g, 4°C), re-suspended in 5 ml ice cold SMP buffer (270 mM sucrose, 1 mM $MgCl_2$, 5 mM sodium phosphate [pH 6.5]), centrifuged (10 min, 8,5000 x g, 4°C) another time and re-suspended in 600 µl ice cold SMP buffer. Of the 600-µl cell suspension 580 µl were transferred into a pre-chilled 0.4 cm electroporation cuvette (Sigma-Aldrich, Dorset, UK) containing 30 µl of 96% (v/v) pure non-denatured ethanol and 0.5 to 5 µg of methylated plasmid DNA. The cell-ethanol-plasmid suspension was incubated for 5 min on ice and, subsequently, electroporated using a Gene Pulser Xcell™ electroporation system (Bio-Rad, Hemel Hempstead, UK), a voltage of 1.8 kV, a capacitance of 25 µF and a resistance of ∞ Ω generating a time constant of 10–18 ms. Immediately after the pulse cells were transferred into pre-warmed 2x YTG broth supplemented with 40 µg/ml uracil, if required, and recovered for 6 to 16 h. Following, cells were centrifuged (10 min, 8,500 x g, RT), re-suspended in 250 µl PBS and plated on RCM agar plates containing the appropriate antibiotic selection (Table 1). Transformants typically took 16 to 24 h (replicative, non-integrative plasmids) or 48 h (ACE, AE) to appear. If transformation efficiencies were to be determined, 100 µl of the 250-µl re-suspension were serial diluted in a total of 1,000 µl PBS of which 100 µl were plated. Colonies were enumeration after a 24-h incubation. All centrifugation steps and washes were carried out anaerobically at 4°C or on ice. If needed, competent cells were frozen until further use. Therefore, cell pellets were re-suspended in 540 µl SMP buffer, supplemented with 60 µl DMSO (10% [v/v]) and frozen at -80 °C.

Segregational stability assay

The segregational stability of the modular plasmids pMTL82151, pMTL83151, pMTL84151 and pMTL85151 was determined as described previously (Heap et al., 2009). Briefly, per modular vector two CRG4111 transformants verified to carry the respective shuttle plasmid,

were inoculated from RCM_{Tm15} plates into 1 ml 2x YTG_{Tm15} broth, grown for 12 h and used to re-inoculate another 1 ml 2x YTG_{Tm15} broth with 1% (v/v) inoculum. The fresh culture was incubated for another 12 h before it was washed three times with PBS, re-suspended in 1 ml PBS and used to inoculate 1 ml of un-supplemented 2x YTG broth at a 1% (v/v) inoculum to start the segregational stability assay. For the assay, the plasmid harbouring cells were sub-cultured every 12 h in un-supplemented 2x YTG broth using a 1% (v/v) inoculum of the preceding culture. Every 24 h twice 100 µl of the well growing culture were serial diluted in PBS until 10^{-8}. Of each dilution 10 µl were spotted twice onto RCM and RCM_{Tm15} agar plates and incubated for 24 h before CFUs were enumerated. The segregational stability was calculated as percent stability per generation using the formula $\sqrt[n]{R}$. Thereby, R represents the portion of cells which still contain the plasmid at the last time point of the experiment, n the number of generations at this time point grown without selection.

Construction of the ACE vectors

ACE *pyrE* KO pMTL-KS01: To generate ACE *pyrE* truncation plasmids on the basis of pMTL-JH12 (Heap et al., 2012) an internal 300-bp fragment of the *C. pasteurianum pyrE* gene (CLPA c2685, Poehlein et al., 2015) lacking the first 34 nt and an 1,200-bp sequence immediately downstream of the *pyrE* gene were PCR-amplified from genomic DNA (Table 1) DNA using the oligonucleotides KS001_SHA12_Fw and KS001_SHA12_Rev or KS002_LHA_Fw and KS002_LHA_Rev (Table S1). The 5' *Sbf*I and 3' *Not*I restriction sites added to the shorter left homology arm (LHA, 300 bp) and the 5' *Nhe*I and 3' *Asc*I restriction sites added to the longer right homology arm (RHA, 1,200 bp) were used to clone the PCR fragments into plasmid pMTL85151 between the *Sbf*I and *Asc*I restriction sites. The resultant plasmid was designated pMTL-KS01, the *pyrE* KO ACE vector (Table 1).

The *pyrE* ACE complementation vector, pMTL-KS12: A transcriptional terminator sequence (that of the *C. tetani* E88 glutamyl-tRNA synthetase gene, $T_{Ctet\ gluRS}$) was inserted proximal to the LHA, through its synthesis as a 77-bp *Sbf*I DNA fragment and insertion into the unique *Sbf*I site of pMTL-KS01 to yield plasmid pMTL-KS02. A Quik Change II Site-Directed Mutagenesis Kit (Agilent Technologies, Stockport, UK) and the oligonucleotides QE_T1Sbf_Fw and QE_T1Sbf_Rev (Table S1) was then used to silence the *Sbf*I recognition site located upstream of $T_{Ctet\ gluRS}$ resulting in the plasmid pMTL-KS03 (Table 1). As the 42-bp *C. pasteurianum* ferredoxin terminator ($T_{Cpa\ fdx}$, Table 1) of plasmid pMTL85151 was lost during the insertion of the RHA, steps were taken to restore its presence. This was accomplished by synthesising a contiguous *Nhe*I/*Asc*I fragment comprising the LHA and $T_{Cpa\ fdx}$ and inserting it between the *Nhe*I and *Asc*I sites of pMTL-KS03 to generate pMTL-KS04 (Table 1). To enable an easy replacement of the RHA without the loss of the $T_{Cpa\ fdx}$, an *Asi*SI recognition site was introduced between the LHA of pMTL-KS04 and the $T_{Cpa\ fdx}$ and the by site-directed mutagenesis and the use of the oligonucleotides QE_AsiSI add_Fw and QE_AsiSI add_Rev (Table S1). The generated plasmid was named pMTL-KS10 (Table 1).

To make the pyrE repair vector pMTL-KS12, a 548-bp fragment of pyrE representing the LHA and lacking only the first 34 nt of pyre is to be swapped with the 300bp LHA fragment of pMTL-KS10. oligonucleotides KS001_SHA12_Fw and KS001_SHA14_Rev were used to PCR amplified the desired DNA fragment from genomic DNA. These oligonucleotides added 5' a *Sbf*I and 3' a *Not*I recognition site to the final PCR product. Following, the modified *Sbf*I and *Not*I equipped PCR fragment was cloned into appropriately digested pMTL-KS10. The result plasmid is pMTL-KS12. The only difference between pMTL-KS10 and pMTL-KS12 is the LHA it carries.

Construction of the Allelic Exchange KO plasmids

Typically, target specific AE cassettes were cloned into pMTL-KS15 using *Sbf*I and *Nhe*I. To enable an alternative cloning strategy in case the AE cassette contains the relative abundant 6-bp *Nhe*I recognition site, an 8-bp *Asi*SI recognition side was introduce between the *Nhe*I recognition site and the terminator $T_{Cpa\ fdx}$ employing the oligonucleotides QE_ AsiSIintro_AA_Fw and QE_ AsiSIintro_AA_Rev (Table S1) and the Quik Change II Site-Directed Mutagenesis Kit (Agilent Technologies, Stockport, UK) according the manufacturer's recommendations. The resulting plasmid was called pMTL-KS16 (Table 1). For both, pMTL-KS15 as well as pMTL-KS16, the insertion of the AE cassette by *Sbf*I and *Nhe*I or *Sbf*I and *Asi*SI lead to a removal of the gratuitous 300-bp *pyrE* SHA-*lacZα* ORF/MCS-'cargo' sequence origination from the plasmid pMTL85151-$T_{Ctet\ gluRS}$-SHA.

AE cassettes for the markerless and in-frame deletion of genes were generated by SOE PCR using the BIO-X-ACTTM Short Mix according to the manufacturer's recommendation (Bioline Reagents, London, UK). A 700-bp region immediately upstream and downstream of the gene of interest including its start and stop codon were amplified employing genomic DNA and target specific oligonucleotide pairs, Fw1 and Rev1 for the amplification of the upstream region and Fw2 and Rev1 for the amplification of the downstream region. Typically oligonucleotides Rev1 and Fw1 are characterised by a 25- to 35-bp long overlapping complementary sequence enabling the later fusion of the two PCR fragments in a third PCR reaction. Oligonucleotides Fw1 and Rev2 equipped the particular PCR fragment with a 5' *Sbf*I and a 3' *Nhe*I restriction site. Following the generation of the two separate PCR fragments, self-same were gel purified and used in equimolar amounts in a third PCR reaction employing the oligonucleotides Fw1 and Rev2. The final PCR product was digested using *Sbf*I and *Nhe*I, purified and cloned into the equally treated AE plasmid pMTL-KS15 resulting in a target specific AE vector. All generated AE plasmids were confirmed by analytic restriction digest and Sanger sequencing.

For the generation of the *spo0A* KO cassette the oligonucleotides KS013_spo0A_Fw1 and KS013_spo0A_Rev1 and KS013_spo0A_Fw2 and KS013_spo0A_Rev2 were employed (Table S1). The final AE vector was called pMTL-KS15::spo0A (Table 1).

For the generation of the hdyA KO cassette the oligonucleotides KS014_hyd_Fw1 and KS014_hyd_Rev1 and KS014_hyd_Fw2 and KS014_hyd_Rev2 were employed (Table S1). The final AE vector was called pMTL-KS15::hydA (Table 1).

For the generation of the rex KO cassette the oligonucleotides KS015_rex_Fw1 and KS015_rex _Rev1 and KS015_rex _Fw2 and KS015_rex _Rev2 were employed (Table S1). The final AE vector was called pMTL-KS15::hydA (Table 1).

For the generation of the dhaBCE KO cassette the oligonucleotides KS016_dhaBCE_Fw1 and KS016_dhaBCE _Rev1 and KS016_dhaBCE _Fw2 and KS016_dhaBCE _Rev2 were employed (Table S1). The final AE vector was called pMTL-KS15::dhaBCE (Table 1).

Complementation plasmid

To verify the observed phenotype of formerly generated KO strains, self-same strains were *in trans* complemented employing ACE and the genomic *pyrE* locus. Typically plasmids for the integration of the homologous and heterologous DNA were constructed as described below. First, the open reading frame encoding the target gene was amplified from genomic DNA using

BIO-X-ACT™ Short Mix (Bioline Reagents, London, UK) and gene specific primers which added a 5' *Not*I and a 3' *Nhe*I recognition site to the PCR fragment. *Not*I and *Nhe*I were used to clone the PCR fragment into the equally digested ACE plasmid. Generally pMTL-KS12 (Table 1) was employed. All generated complementation vectors were verified by a diagnostic restriction digest and Sanger sequencing. The homologous or heterologous genes was cloned either with its own promoter or with a heterologous promoter inserted into the plasmid pMTL-KS12 by *Not*I and *Nde*I restriction digest.

For the specific task of generating a *spo0A* complementation vector, the indigenous *C. pasteurianum spo0A* gene (CLPA c19180, Poehlein et al., 2015) as well as a 267-bp sequence immediately upstream of the *spo0A* start codon predicted to comprise the promoter region of *spo0A* (SoftBerry BPROM software, Solovyev and Salamov, 2011) were amplified from genomic DNA using the oligonucleotides KS004_spo0A_compl_Fw and KS004_spo0A_compl_Rev (Table S1). Subsequently, the 1095-bp fragment was cloned into pMTL-KS12 and confirmed as described above. The complementation plasmid pMTL-KS12::*spo0A* was used to generate the *C. pasteurianum spo0A* complementation (repair) strain CRG5518 as described in Methods.

The *rex* complementation vector pMTL-KS12::*rex* was constructed by amplifying a 816-bp fragment with primers KS010_rex_compl_Fw/KS010_rex_compl_Rev (Table S1) comprising the 183-bp sequence upstream of *rex* and the *rex* gene from *C. pasteurianum DSM 525* and cloning it into the *Not*I/*Nhe*I recognition sites of pMTL-KS12. The plasmid was used to generate strain *C. pasteurianum* DSM525-H1::*rex** (CRG5524) (Table 1).

The plasmid for complementation of the hydrogenase *hyd* was constructed by amplifying a 2118-bp fragment comprising the 393-bp sequence upstream of *hyd* and the *hyd* gene with primers KS011_hyd_compl_Fw/ KS011_hyd_compl_Rev (Table S1) which was cloned into the *Not*I/*Nhe*I recognition sites of pMTL-KS12 leading to plasmid pMTL-KS12::*hyd.* The plasmid was used to generate strain *C. pasteurianum* DSM525-H1::*hydA** (CRG5530) (Table 1).

Finally, the *dhaBCE* complementation plasmid *C. pasteurianum* DSM525-H1::*dhaBCE** was constructed by amplifying a 2984-bp fragment comprising the 294-bp sequence upstream of dhaB and the dhaBCE genes with primers KS012_dhaBCE_compl_Fw/ KS012_dhaBCE_compl_Rev (Table S1)and cloning the *Not*I/*Nhe*I recognition sites of pMTL-KS12. The plasmid was used to generate strain *C. pasteurianum* DSM525-H1::*dhaBCE** (CRG5536) (Table 1).

Figures

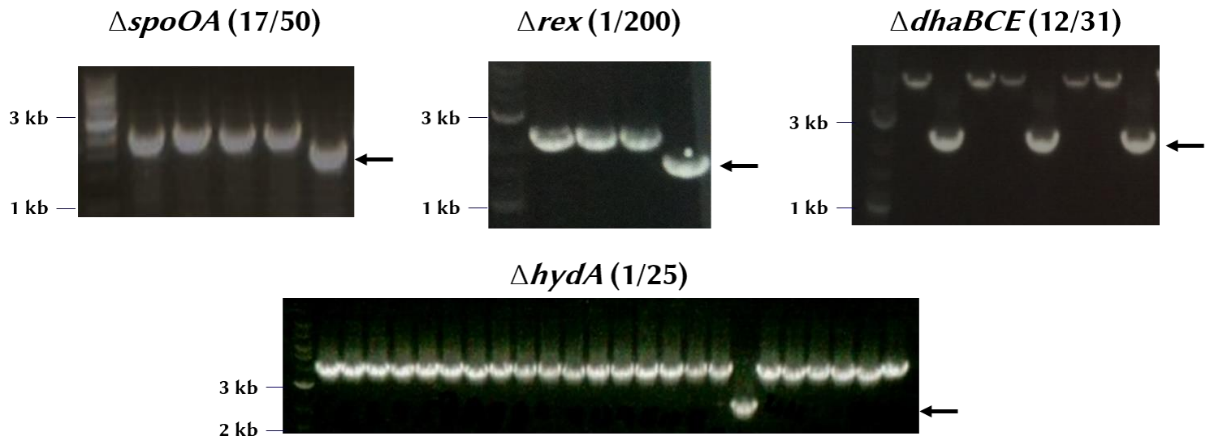

Fig. S1. PCR of double-crossovers after allelic exchange knock out of target genes. Ratios of successful knock outs over wild type revertants are given for each target.

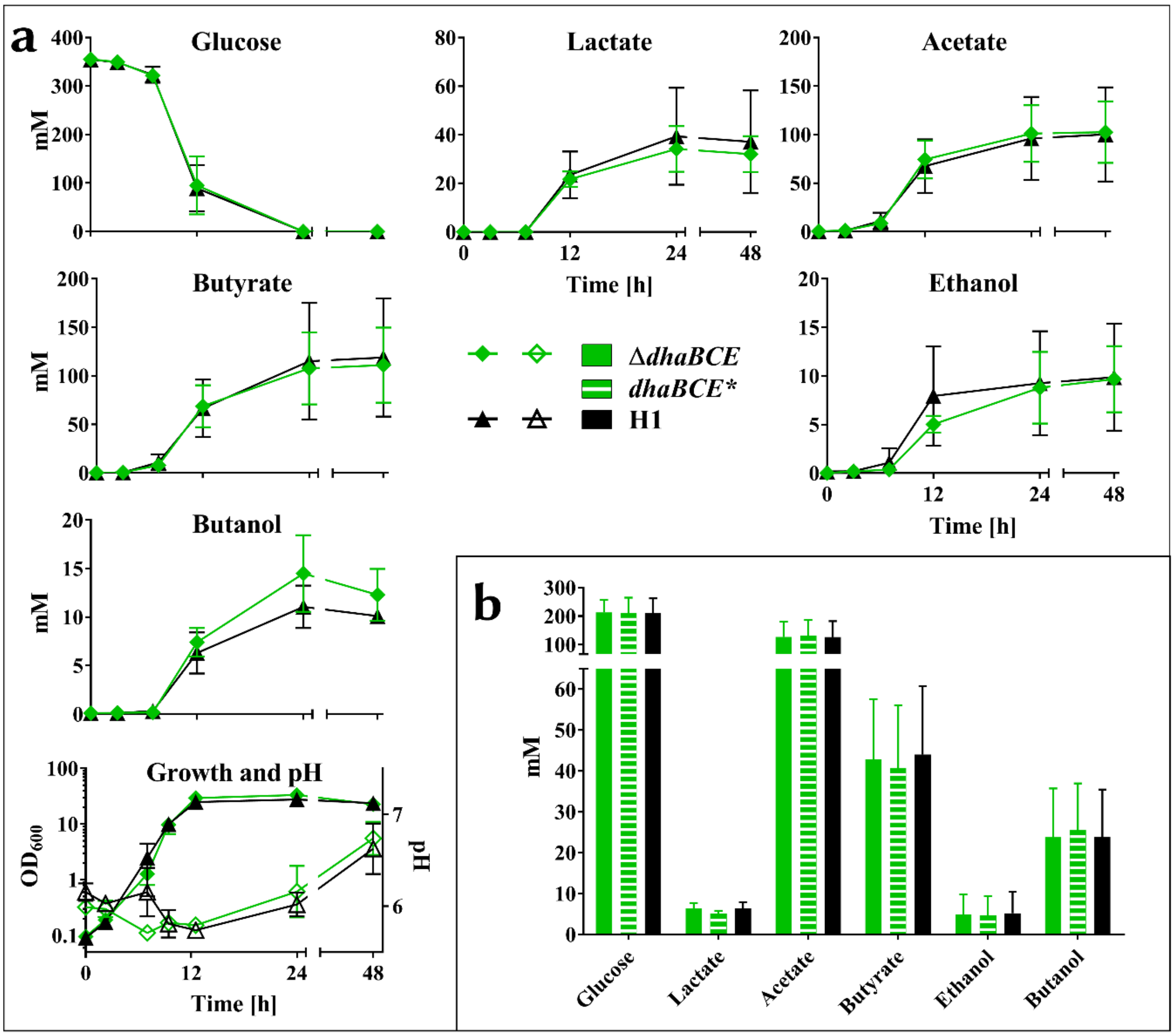

Fig. S2. Glucose fermentations of *C. pasteurianum*-H1 (H1), *C. pasteurianum*-H1Δ*dhaBCE* (Δ*dhaBCE*) and *C. pasteurianum*-H1-*dhaBCE* complementation (*dhaBCE**). a) Bioreactor fermentation with 60 g/l glucose in Biebl medium at pH 6 was carried out for 48 h with fermentation being visibly completed after 24 hours. Error-bars indicate range of two fermentations. b) Histogram showing product formation of serum bottle fermentation of deletion strains and complementations. Glucose usage and product formation is shown after 24 h. Error-bars indicate standard error of three replicates.

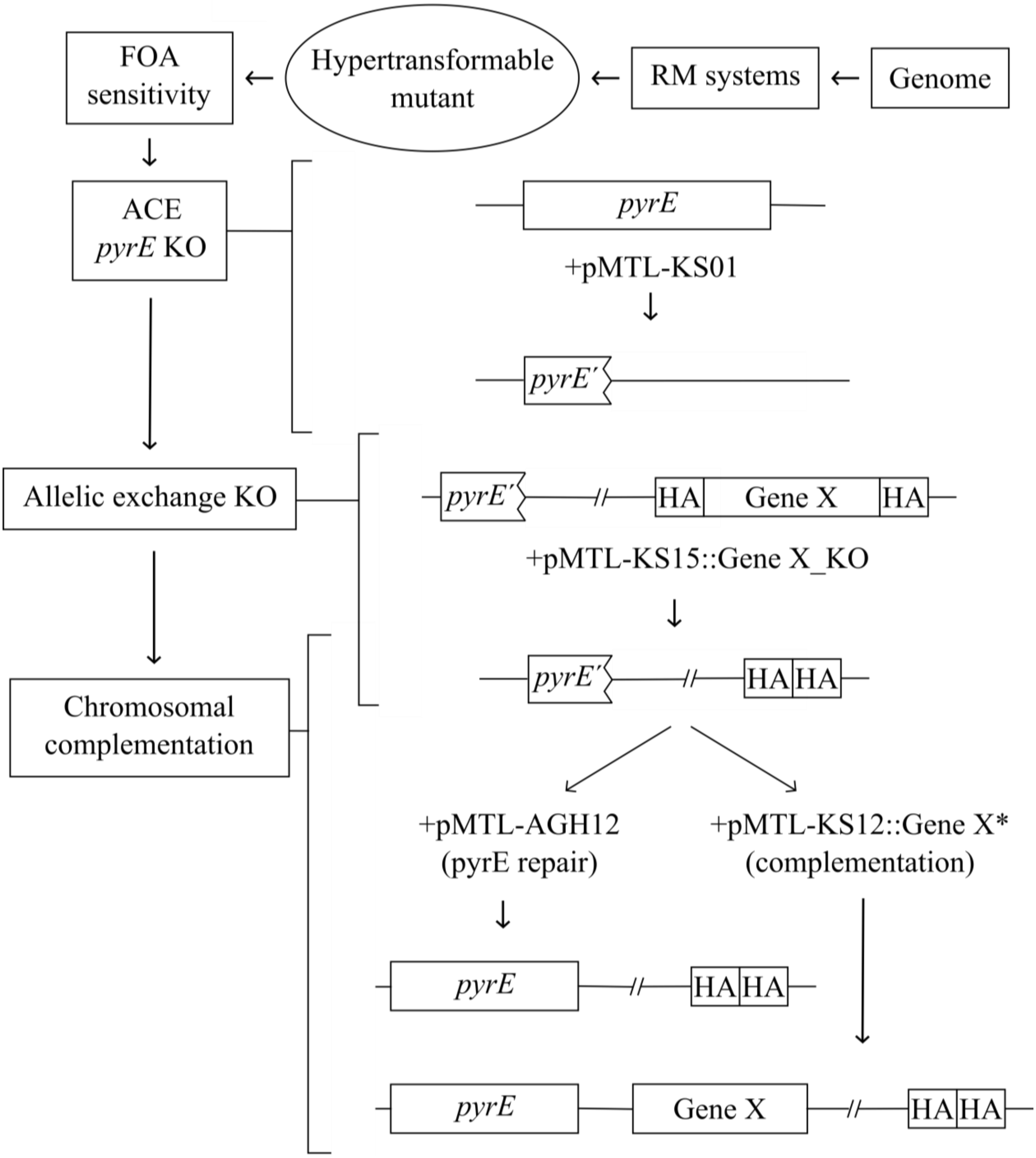

Fig S3. The clostridial roadmap implemented in *C. pasteurianum* (Minton et al., 2016). The genome was published by Poehlein et al. (2015) and restriction modification systems were identified. Plasmid DNA was protected against restriction by *in vitro* methylation with *E. coli* carrying plasmid pMTL-CR1 expressing the M.BepI methylase. Due to low transformation efficiency in the wild type a screen was done for hypertransformable mutants (not in the original roadmap, indicated by oval). 5-fluorouracil (FOA) sensitivity was assayed based on which a *pyrE* truncation strain (*pyrE*′) was produced with the ACE plasmid pMTL-KS01. This strain allowed the use of allelic exchange technology to make knock-outs of genes of interest (Gene X) guided by homology arms (HAs) up- and downstream of the gene. Knock-out mutants were complemented at the *pyrE* locus by repairing the *pyrE* allele and supplying the gene with its native promoter downstream of *pyrE* using plasmids pMTL-KS12::Gene X*. The *pyrE* allele was repaired to wild type without additional cargo with plasmid pMTL-AGH12.

Tables

Table S1. Primers used in this study. *Upper case letters: added restriction endonuclease recognition sites; Italics: 5' overhang complementary sequences in SOE PCR; Underlined: Side-directed mutagenesis sites.

Name	Sequence 5' to 3'*
PCR	
KS001_SHA12_Fw	cccgggCCTGCAGGtaaaagagtcagaggctcttttagaaggacattttttac
KS001_SHA12_Rev	cccgggGCGGCCGCccttatctcctttttaacttcaaatcctcttttaatagtc
KS001_SHA14_Rev	cccgggGCGGCCGCtcaaactatatttctgctgccaggttttatgtatggtata
KS002_LHA_Fw	gGCTAGCtttatcaaatatttttagtataattgactttatttaaaacataattgtttgatttgtaattgttg
KS002_LHA_Rev	cccgggGGCGCGCCaagctactttctgctacagagtaagttttatagggata
KS003_85151_LHA_Rev2	gcaccccctgaacccccatgct
KS004_Cpa_pyrE_gen_Fw	gttttacatgaaatatttaagaatttttggctgctataagc
KS004_Cpa_pyrE_gen_Rev	catggactacaatagcttcataataaccgcc
KS005_spo0A_genome_Fw	ccaataacagatgttgatactggtacaatgctcag
KS005_spo0A_genome_Rev	tctagaaaaccatcttccgaaaaagataccaaacatactt
KS006_rex_genome_Fw	ggctgtccaaattttgccttagtagaagctatt
KS006_rex_genome_Rev	cccttataagctataagatactgttcgagccattc
KS007_hyd_genome_Fw	gatggtactaataatgttaagctaattgatgatagtattatagatgg
KS007_hyd_genome_Rev	gcaactgatttcaatattaatatgtctcatagtgaaaaatcc
KS008_bcd_genome_Fw	gaatggattaaaaaatagtttggggtggaaaatatgactg
KS008_bcd_genome_Rev	gttcttgcaaatatagaaggatcaaaatgctcctc
KS009_spo0A_compl_Fw	cccgggGCGGCCGCaataaaagactttagagtaaattctaaagtcttttataattaatgttaaaataaattattatag
KS009_spo0A_compl_Rev	cccgggGCTAGCctactgagccacattcttaaccttatttttaagtctaag
KS010_rex_compl_Fw	cccgggGCGGCCGCaatttaatcaccagcctaatattaagtg
KS010_rex_compl_Rev	cccgggGCTAGCttatattgtcttattcattaggcaagtc
KS011_hyd_compl_Fw	cccgggGCGGCCGCgtaaattttattttcaatttctatatatgattattaaatcctcacaattaatgc
KS011_hyd_compl_Rev	cccgggGCTAGCttatttttttatatttaaagtgtaatatttcatgggcacgacc
KS012_dhaBCE_compl_Fw	cccgggGCGGCCGCtttttttaaagactaatgctataaaaagtaggcaagataattagaattattttaattatattg
KS012_dhaBCE_compl_Rev	cccgggGCTAGCctagtcctctattctaactttatttctcttactgtatac
AGH0001_pyrE_Fw	cccgggCCTGCAGGtaaaagagtcagaggctcttttagaaggaca
AGH0002_pyrE_Rev	cccgggGGCGCGCCaagctactttctgctacagagtaagttttat
SOE-PCR	
KS013_spo0A_Fw1	aaaaaCCTGCAGGtgtaaatgaaaataatcccatggg
KS013_spo0A_Rev1	ccctaaaactactctcaacccacctacattttttaatactccccttttatatttctattc
KS013_spo0A_Fw2	gaatagaaatataaaggggagtattaaaaatgtaggtgggttgagagtagttttaggg
KS013_spo0A_Rev2	aaaaaGCTAGCtcaaatcaaataaatttacagcagatga
KS014_hyd_Fw1	aaaaaCCTGCAGGatcaatttaatatcaatgaaaaaaatatatactattatacttccataaataatagtgg
KS014_hyd_Rev1	*cttaaaattaaatctaggaggctagatatg*taaatttattatttgaaaataaaaataaaaacagcattatgaaaatattgtttttttcataatgctg
KS014_hyd_Fw2	*gtttttatttttattttcaaataataaattta*catatctagcctcctagatttaattttaagatctttatttaatttaacagc
KS014_hyd_Rev2	cccgggGCTAGCataaatatgaataatataatacaagtaaaaggaataacttataaacttaatgatcttatac
KS015_rex_Fw1	cccgggCCTGCAGGtttcaagttttctgaacaccttattacctagtattccaaattttc
KS015_rex_Rev1	*tttcagaggtaaggggtgcaacagttg*taaagatttcacattcatatttaatactgctaaaatacaaaagctattattataag
KS015_rex_Fw2	*gtattaaatatgaatgtgaaatcttta*caactgttgcaccccttacctctgaaatatat

KS015_rex_Rev2	cccgggGCTAGCgatttataattttatcataattttctgaggaaaatccaagtccatttaaaac
KS016_dhaBCE_Fw1	cccgggCCTGCAGGgtctgtgatttatcggagattctcagatatacactaa
KS016_dhaBCE_Rev1	*aatttcataatttccacctgccaacta*catatataatcctcctttatcaaatctctgttgttcttatc
KS016_dhaBCE_Fw2	*agatttgataaaggaggattatatatg*tagttggcaggtggaaattatgaaattcgttgct
KS016_dhaBCE_Rev2	cccgggGCTAGCgcaggtattcttctttcctgcacatcacc
Side-directed mutagenesis	
QE_T1Sbf_Fw	cgacgagcaaggcaagaccgatcgggccccctcctggctgccaatagataaaataaagtctgccac
QE_T1Sbf_Rev	gtggcagactttattttatctattggcagccaggagggggcccgatcggtcttgccttgctcgtcg
QE_AsiSI add_Fw	atttgcaggcttcttatttttatgcgatcgctttatcaaatatttttagtataattgactttatttaaaacataattgtttgatttgtaattgttg
QE_AsiSI add_Rev	caacaattacaaatcaaacaattatgttttaaataaagtcaattatactaaaaatatttgataaagcgatcgcataaaaataagaagcctgcaaat
QE_ AsiSIintro_AA_Fw	caacagttgcgcagcctgaatggcgaatggcgctagcgcgatcgcataaaaataagaagcctgcatttgcaggcttcttatt
QE_ AsiSIintro_AA_Rev	aataagaagcctgcaaatgcaggcttcttatttttatgcgatcgcgctagcgccattcgccattcaggctgcgcaactgttg

Table S2. Table of putatively Rex regulated genes with target sequence and distance from closest start codon.

Genome position	Sequence	Locus tag	Gene	Protein function	ATG-distance	Gene on
185124	TTGATAAATTT TTAATAA	CLPA_c01790	*mpg1*	putative mannose-1-phosphate guanyltransferase	84	complement
193348	TTATTATAAAT ATAACAA	CLPA_c01840		transcriptional regulator	81	complement
442350	TTGTTATGTAC TTAACAA	CLPA_c04110		transcriptional regulator, AraC family	119	same strand
470707	TTGTTATATTT ATAACAT	CLPA_c04410		hypothetical protein	146	same strand
542964	TTGTTAAATTG TTAACAA	CLPA_c05110		iron (metal) dependent repressor, DtxR family	377	same strand
580610	TTGTTATATAA ATAACAA	CLPA_c05450		nitroreductase	413	complement
617573	TTGTTAAATAA ATAACAA	CLPA_c05730	*adhE1*	aldehyde-alcohol dehydrogenase	37	same strand
946770	TAGTTAAAAAT TTAACAT	CLPA_c08620	*thlA1*	acetyl-CoA acetyltransferase	114	complement
955099	TTGTTAATACA CTAACAG	CLPA_c08710		MarR family transcriptional regulator	15	same strand
1058613	TTTTTATTTTA TTGACAA	CLPA_c09900		nicotinamidase family protein	74	same strand
1324031	TTGATAAAATA CTAACAT	CLPA_c12290		ABC-type Fe3+-siderophore transport system, permease component	99	same strand
1351981	TTATTATAAAA TTAACAA	CLPA_c12470	*cbiM*	cobalt transport protein CbiM	270	complement
1390593	TTTTTATTATA TTAACAT	CLPA_c12900		hypothetical protein	498	complement
1393572	TTATTACAATA TTAACAA	CLPA_c12920		peptidase family M50	71	same strand
1425190	TTGATAGTGTA TTAAAAA	CLPA_c13250	*cysE*	serine acetyltransferase	568	same strand
1429806	TTGTTAAAAAT TTGACAA	CLPA_c13290		hypothetical protein	86	same strand
1429918	TTTTTATATCA ATAACAA	CLPA_c13300		hypothetical protein UPF0178	221	same strand
1615045	TTATTAGAAAA ATAACAA	CLPA_c15130		hypothetical protein	35	same strand
1617865	TTGCTAATTAT TTAACAT	CLPA_c15160	*adhE2*	aldehyde-alcohol dehydrogenase	97	complement
1881969	TTGATAATTAT TTAACAA	CLPA_c17480		flavodoxin	83	same strand
1952030	TTATTAAATAA TTGACAA	CLPA_c18120	*flgB*	flagellar basal body rod protein FlgB	428	complement
2225549	TTGATAAACAA ATAACAT	CLPA_c20830	*pta*	phosphate acetyltransferase	256	same strand
2450806	TTGATAATATT TTAACAA	CLPA_c22810	*dhaB*	glycerol dehydratase large subunit	64	complement

2545028	TTGATAATTTT TTAACAT	CLPA_c23760	*aldA*	putative aldehyde dehydrogenase AldA	81	complement
2563784	TAGATAATTTT TTAACAA	CLPA_c23901		hypothetical protein	307	same strand
2757266	TTTATACACTC TTAACAA	CLPA_c25790		hypothetical protein	173	complement
2776821	TTGTTTAAAAA CTAACAA	CLPA_c26000		NifU-like domain containing protein	121	same strand
2843829	TTATTATACTA TTAACAT	CLPA_c26620		hypothetical protein	82	complement
2937664	TTGTTAATATA TTAACAT	CLPA_c27550		LysR family transcriptional regulator	75	same strand
2984508	TTATTAATTAA TTAAAAA	CLPA_c27880		putative transketolase N-terminal section	156	same strand
3015230	TAGGTAAAGTA TTAACAA	CLPA_c28170	*gpmI*	2,3-bisphosphoglycerate-independent phosphoglycerate mutase	162	same strand
3067448	TTGTTAGAATA TTAACAA	CLPA_c28630	*crt2*	3-hydroxybutyryl-CoA dehydratase	27	same strand
3092582	TTGATAATATA TTAAAAA	CLPA_c28840	*mtlA*	PTS system mannitol-specific EIICB component	307	same strand
3106226	TTGTTTGACAA TTAACAA	CLPA_c28930	*adhE4*	aldehyde-alcohol dehydrogenase	121	same strand
3106321	TAGTTAAATTT TTAACAA	CLPA_c28930	*adhE4*	aldehyde-alcohol dehydrogenase	216	same strand
3259481	TTATTAATGGA TTAACAA	CLPA_c30260	*ssuC1*	putative aliphatic sulfonates transport permease protein SsuC	216	same strand
3612217	TTTTTAATTCA TTGACAA	CLPA_c33470		hypothetical protein	365	same strand
3634909	TTTTTAAAGGT TTAACAA	CLPA_c33660	*mdeA3*	methionine gamma-lyase	44	same strand
3707143	TTGCTAGTTTA TTAACAT	CLPA_c34300		hypothetical protein	17	complement
3835308	TTGTTATTTAA TTCACAA	CLPA_c35710	*thlA2*	acetyl-CoA acetyltransferase	47	complement
3846267	TATTTAAAATA TTAACAA	CLPA_c35810	*spoVD2*	stage V sporulation protein D	207	same strand
3971344	TTTTTAGAAAT ATAACAA	CLPA_c37090		hypothetical protein	34	same strand
4077063	TTGCTAATTAA TTAATAA	CLPA_c38200	*nifH6*	nitrogenase iron protein	813	same strand
4082067	TTGTTATTGTA TTAAAAA	CLPA_c38230	*opuCA*	carnitine transport ATP-binding protein OpuCA	111	same strand
4160172	TTGCTAAAAAT TTAAAAA	CLPA_c39050	*asd*	aspartate-semialdehyde dehydrogenase	589	same strand
4166771	TTATTAATTTA TTAAAAA	CLPA_c39090		NAD(P)H dehydrogenase	136	same strand
4264175	TTGTTAATATA TTTACAA	CLPA_c40020		hypothetical protein	14	same strand

References

Heap, J.T., Pennington, O.J., Cartman, S.T. and Minton, N.P., 2009. A modular system for Clostridium shuttle plasmids. *Journal of microbiological methods*, *78*(1), pp.79-85.

Heap, J.T., Ehsaan, M., Cooksley, C.M., Ng, Y.K., Cartman, S.T., Winzer, K. and Minton, N.P., 2012. Integration of DNA into bacterial chromosomes from plasmids without a counter-selection marker. *Nucleic acids research*, *40*(8), pp.e59-e59.

Minton, N.P., Ehsaan, M., Humphreys, C.M., Little, G.T., Baker, J., Henstra, A.M., Liew, F., Kelly, M.L., Sheng, L., Schwarz, K. and Zhang, Y., 2016. A roadmap for gene system development in *Clostridium*. Anaerobe 41, 104-112.

Poehlein, A., Grosse-Honebrink, A., Zhang, Y., Minton, N.P. and Daniel, R., 2015. Complete genome sequence of the nitrogen-fixing and solvent-producing Clostridium pasteurianum DSM 525. *Genome announcements*, *3*(1), pp.e01591-14.

Solovyev, V. and Salamov, A., 2011. Automatic annotation of microbial genomes and metagenomic sequences. *Metagenomics and its applications in agriculture, biomedicine and environmental studies*, pp.61-78.
